# Integrated *In Silico* Analysis of Pathway Designs for Synthetic Photo-Electro-Autotrophy

**DOI:** 10.1371/journal.pone.0157851

**Published:** 2016-06-23

**Authors:** Michael Volpers, Nico J. Claassens, Elad Noor, John van der Oost, Willem M. de Vos, Servé W. M. Kengen, Vitor A. P. Martins dos Santos

**Affiliations:** 1 Laboratory of Systems and Synthetic Biology, Wageningen University, Dreijenplein 10, 6703 HB, Wageningen, The Netherlands; 2 LifeGlimmer GmbH, Markelstr. 39a, 12136, Berlin, Germany; 3 Laboratory of Microbiology, Wageningen University, Dreijenplein 10, 6703 HB, Wageningen, The Netherlands; 4 Institute of Molecular Systems Biology, ETH Zürich, Auguste-Piccard-Hof 1, 8093, Zürich, Switzerland; 5 Department of Bacteriology and Immunology, Helsinki University, Haartmaninkatu 3, 00014, Helsinki, Finland; University of Freiburg, GERMANY

## Abstract

The strong advances in synthetic biology enable the engineering of novel functions and complex biological features in unprecedented ways, such as implementing synthetic autotrophic metabolism into heterotrophic hosts. A key challenge for the sustainable production of fuels and chemicals entails the engineering of synthetic autotrophic organisms that can effectively and efficiently fix carbon dioxide by using sustainable energy sources. This challenge involves the integration of carbon fixation and energy uptake systems. A variety of carbon fixation pathways and several types of photosystems and other energy uptake systems can be chosen and, potentially, modularly combined to design synthetic autotrophic metabolism. Prior to implementation, these designs can be evaluated by the combination of several computational pathway analysis techniques. Here we present a systematic, integrated *in silico* analysis of photo-electro-autotrophic pathway designs, consisting of natural and synthetic carbon fixation pathways, a proton-pumping rhodopsin photosystem for ATP regeneration and an electron uptake pathway. We integrated Flux Balance Analysis of the heterotrophic chassis *Escherichia coli* with kinetic pathway analysis and thermodynamic pathway analysis (Max-min Driving Force). The photo-electro-autotrophic designs are predicted to have a limited potential for anaerobic, autotrophic growth of *E*. *coli*_,_ given the relatively low ATP regenerating capacity of the proton pumping rhodopsin photosystems and the high ATP maintenance of *E*. *coli*. If these factors can be tackled, our analysis indicates the highest growth potential for the natural reductive tricarboxylic acid cycle and the synthetic pyruvate synthase–pyruvate carboxylate -glyoxylate bicycle. Both carbon fixation cycles are very ATP efficient, while maintaining fast kinetics, which also results in relatively low estimated protein costs for these pathways. Furthermore, the synthetic bicycles are highly thermodynamic favorable under conditions analysed. However, the most important challenge identified for improving photo-electro-autotrophic growth is increasing the proton-pumping rate of the rhodopsin photosystems, allowing for higher ATP regeneration. Alternatively, other designs of autotrophy may be considered, therefore the herein presented integrated modeling approach allows synthetic biologists to evaluate and compare complex pathway designs before experimental implementation.

## 1. Introduction

One of the current grand societal and technological challenges is to establish sustainable production processes for chemicals and fuels. The current biotechnological production is based on a series of relatively inefficient steps, including plant photosynthesis, harvesting and transport of biomass from fields to a biorefinery, pre-treatment of crude biomass and subsequent microbial fermentation [1]. A potentially more efficient production process is offered by autotrophic microorganisms directly converting carbon dioxide and sustainable energy, such as light and electricity, into products. However, many autotrophic microorganisms are not genetically amenable or for other reasons not suitable for such industrial applications. Major advances in synthetic biology start to allow engineering of complex features related to autotrophy into heterotrophic chassis microorganisms, such as *Escherichia coli*. Recently, features such as (partial) carbon fixation pathways [2–8], assimilation pathways of one-carbon compounds [9,10], photosystems [11,12] and direct electron transfer [13,14] have been successfully introduced in *E*. *coli* and some other heterotrophic hosts. However, complete synthetic autotrophy engineered in a heterotrophic host has not yet been demonstrated.

**Fig 1:**
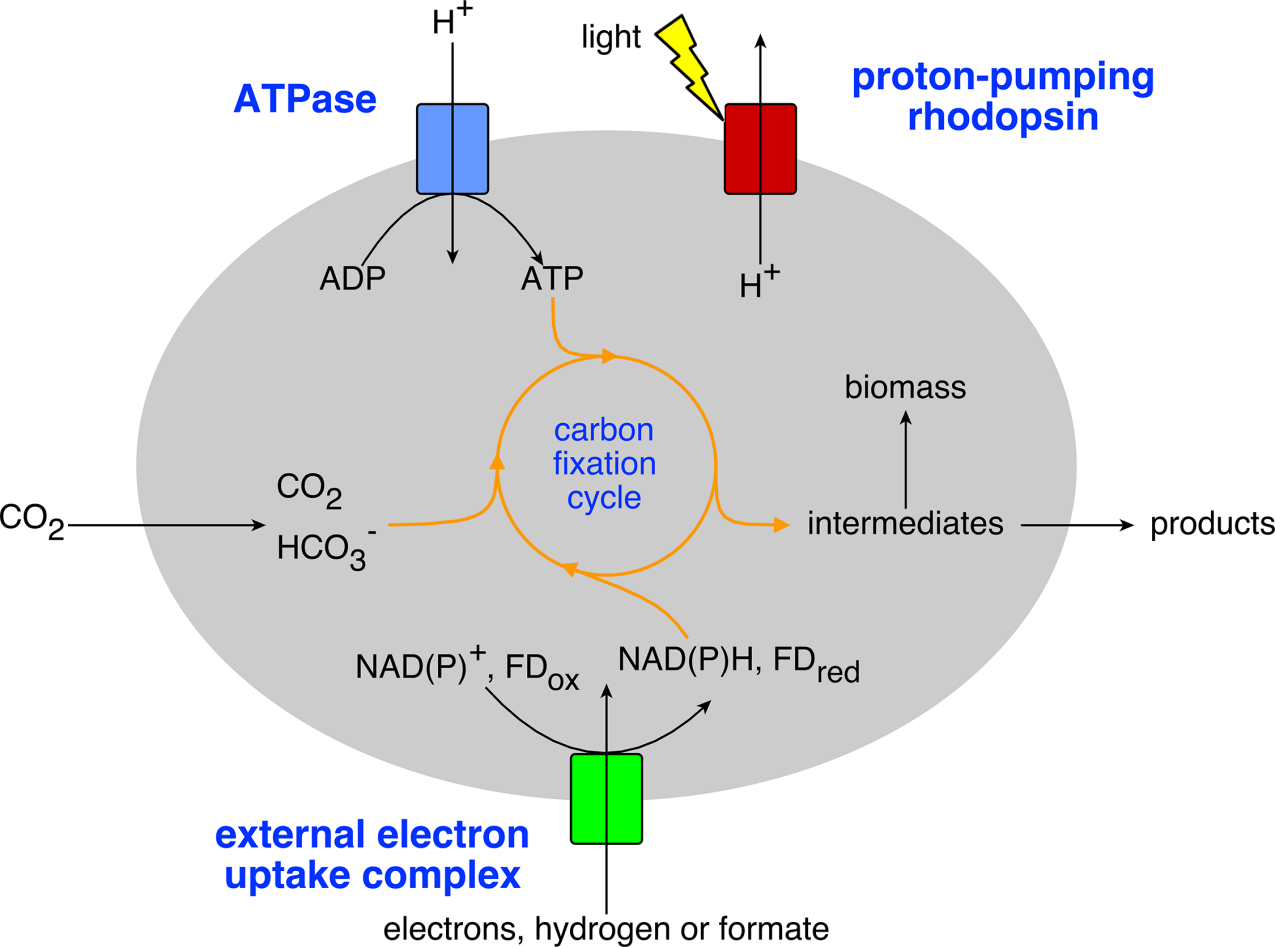
General principle of the photo-electro-autotrophic *E*. *coli*. Key components are a carbon fixation cycle, a mechanism to take up external electrons and the proton-pumping rhodopsins photosystem.

Completely autotrophic, synthetic microorganisms require integration of subsystems for both carbon fixation and energy uptake to regenerate electron donors and ATP. Those subsystems need to be integrated into properly evaluated designs before going into the challenging, time-consuming and expensive process of experimental implementation. Here we present an *in silico* analysis of different designs for so called anaerobic photo-electro-autotrophy in *E*. *coli*. These designs consist of both uptake pathways for electron donors and photosystems to harvest light energy (Fig 1). Electron donor uptake can be achieved through uptake pathways for e.g. low potential electrons from a cathode, formate or hydrogen for regenerating intracellular electron donors, known as microbial electrosynthesis. Most pathways for electrosynthesis function best anaerobically, however, this mostly results in an ATP limitation [1,15–19]. Therefore we included in our designs a proton-pumping rhodopsin (PPR), which is a photosystem that can generate a proton motive force and hence regenerate ATP under anaerobic conditions [11]. These proton-pumping rhodopsin photosystems are less complex to engineer by synthetic biology tools than the common water-splitting reaction-center photosystems [11]. However, contrary to reaction-center photosystems, proton-pumping rhodopsins cannot regenerate electron donors, so for autotrophic growth they need to be complemented with electron donor uptake mechanisms. These electron uptake mechanisms and photosystems could be integrated with a carbon fixation pathway that can be chosen from a variety of (anaerobic) synthetic and natural pathways [20].

Here we investigate the feasibility of the different designs for synthetic photo-electro-autotrophy by using an integrated *in silico* analysis approach. Several *in silico* tools enabled the analysis of key performance factors such as growth rates, pathway thermodynamics, pathway kinetics and systems protein burden. We used the Max-min Driving Force (MDF) to determine the thermodynamic feasibility of pathways [21–24]. We used Flux Balance Analysis (FBA) [25] and Flux Variability Analysis (FVA) [26] to predict growth yields and integrated these data with enzyme and pathway kinetic parameters [20] to predict the protein burden on the cell of the components of the photo-electro-autotrophic designs. Some of these tools have been applied before to analyze carbon fixation pathways and other integrated autotrophic designs [15,17,20,27–30]. Here we combine all of these state-of-the-art *in silico* analysis tools with input of available experimental data to analyze novel photo-electro-autotrophic designs.

The integrated *in silico* analysis allowed us to compare the feasibility of different designs and identify key bottlenecks for realizing photo-electro-autotrophy in e.g. *E*. *coli*. Photo-electro-autotrophic growth in *E*. *coli* under anaerobic conditions, seems drastically limited by ATP. The assumed ATP regeneration through the rhodopsin photosystems is too limited to achieve high growth rates. Improving rhodopsin photosystems pumping rates and/or levels, or alternative ATP regeneration systems have to be considered before successful implementation of the envisioned photo-electro-autotrophic designs in *E*. *coli*. Given the limitations of rhodopsin photosystems, reasonably high photo-electro-autotrophic growth rates above 0.1 h^-1^ are only feasible when assuming that the Non-Growth-Associated Maintenance (NGAM), of *E*. *coli* can be drastically decreased to ~1 mmol ATP/gCDW/h^-^. Furthermore, ATP-efficient carbon fixation cycles, such as the natural reductive TCA (rTCA) cycle and synthetic pyruvate synthase–pyruvate carboxylase–glyoxylate (PyrS-PyrC-Glx) bicycle, result in higher predicted growth rates, while giving relatively low protein costs. Generally, the herein presented, integrated *in silico* approach allows synthetic biologists and metabolic engineers to better evaluate and compare complex designs for e.g. autotrophy before experimental implementation.

## 2. Methods

### 2.1. Autotrophy subsystems for the designs

#### 2.1.1 Carbon fixation pathways and reactions

Carbon fixation is a central part of any autotrophic system. From the six known natural pathways for carbon fixation and a large theoretical repertoire of synthetic pathways for carbon fixation we analyzed six pathways, which are listed in Table 1 with their most important characteristics. Firstly we included the naturally dominant, oxygen-tolerant (i) Calvin cycle (S1 Fig). However, for the photo-electro-autotrophic designs we focused on anaerobic conditions. This enabled us to include carbon fixation pathways containing oxygen-sensitive ferredoxin-oxidoreductase enzymes, which is advantageous, as these pathways are often both kinetically fast and ATP-efficient [20]. Therefore we selected the natural, anaerobic (ii) rTCA cycle (S2 Fig) [31,32].

**Table 1:** General characteristics of the analyzed carbon fixation pathways.

Carbon fixation pathway	Origin pathway	Oxygen sensitive enzymes	Electron donors	Carbon species	Total number of enzymes in cycle*	References, figures
Calvin cycle (reductive pentose phosphate pathway)	Natural, e.g. plants, cyanobacteria	no	NADPH	CO_2_	12	(Berg, 2011), S1 Fig
rTCA cycle (reductive tricarboxylicacid cycle)	Natural, many anaerobic bacteria	yes**	ferredoxin_red_NADPHNADH	CO_2_	8	(Berg, 2011), S2 Fig
3HP-4HB cycle (3-hydroxypropionate -4-hydroxybutyrate cycle)	natural, *aerobic crenarchaeota*	no***	NADPH	HCO_3_^-^	13	(Berg, 2011), S3 Fig
DC-4HB cycle (dicarboxylate-4-hydroxybutyrate cycle)	natural, *anaerobic crenarchaeota*	yes	ferredoxin_red_NADPH	CO_2_, HCO_3_^-^	14	(Berg, 2011) S4 Fig
PyrS-PyrC-Glx bicycle (pyruvate synthase-pyruvate carboxylase-glyoxylate bicycle)	synthetic	yes	ferredoxin_red_NADH	CO_2_, HCO_3_^-^	10	(Bar-Even et al., 2012, 2010), Fig 2
PyrS-PEPC-Glx bicycle (pyruvate synthase-phosphoenol pyruvate carboxylase-glyoxylate bicycle)	synthetic	yes	ferredoxin_red_NADH	CO_2_, HCO_3_^-^	11	(Bar-Even et al., 2012, 2010), Fig 2

*the number of enzymes includes all enzymes which are part of the CO_2_ fixation cycles, note that for the synthetic cycles we also included the number of enzymes for the required glyoxylate assimilation cycle, which consists of 5 additional enzymes

**some natural bacteria harboring the rTCA cycle are microaerobic, they have some mechanism to protect the ferredoxin-oxidoreductases from oxygen or have oxygen-tolerant variants of those [31]

***the natural 3HP-4HB cycle is aerobic, however the variant modelled in this paper contains pyruvate synthase, an oxygen sensitive ferredoxin-oxidoreductase

Furthermore we selected the natural (iii) 3-hydroxypropionate– 4-hydroxybutyrate (3HP-4HB) cycle (S3 Fig); in our analyses, in contrast to the natural aerobic situation for this cycle, the primary product acetyl-CoA is assimilated into the central metabolism using oxygen-sensitive pyruvate synthase. This anaerobic alternative is a more ATP-efficient alternative than the natural assimilation via succinyl-CoA [33]. Even more importantly, as in *E*. *coli* the succinate dehydrogenase generates ubiquinol, which can be only be regenerated to ubiquinone with a high potential electron acceptor, such as oxygen, which is not present in envisioned anaerobic conditions. Furthermore, we note that the 3-HP-4HB cycle we analyzed is the crenarchaeal version, and not the recently elucidated, more ATP-efficient, thermodynamically less favorable, thaumarchaeal verion [34]. While analyzing the 3HP-4HB cycle in *E*. *coli* we identified a more promising alternative cycle, being the natural, anaerobic (iv)–dicarboxylate-4-hydroxybutyrate (DC-4HB) cycle (S4 Fig).

**Fig 2:**
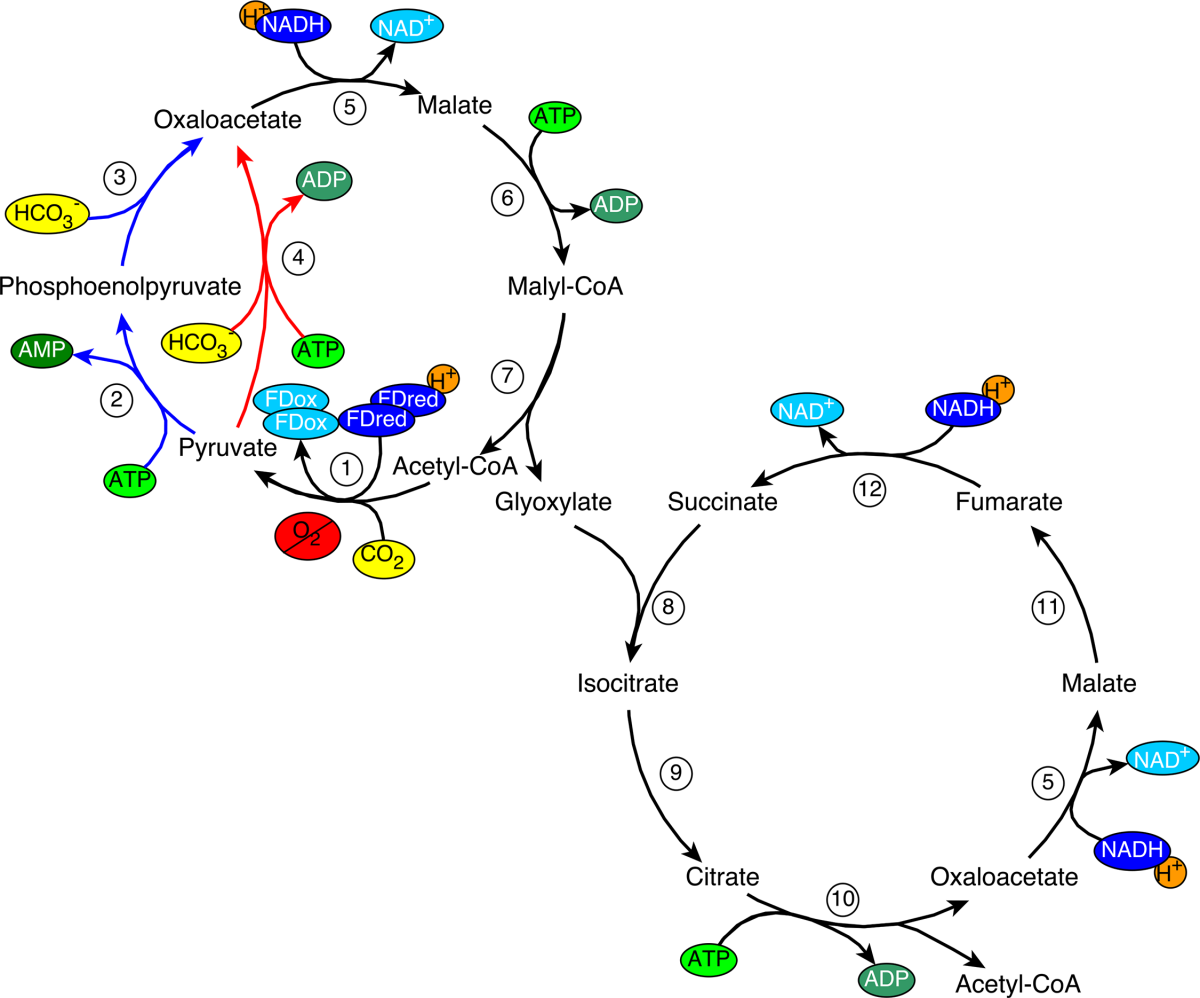
Synthetic carbon fixation pathways: PyrS-PyrC-Glx bicycle (red and black) and the PyrS-PEPC-Glx bicycle (blue and black). Enzymes involved in the bicycles: 1) pyruvate synthase (oxygen sensitive), 2) pyruvate water dikinase, 3) phosphoenolpyruvate carboxylase, 4) pyruvate carboxylase, 5) malate dehydrogenase, 6) malate thiokinase, 7) malyl-CoA lyase, 8) isocitrate lyase, 9) aconitate hydratase, 10) ATP citrate lyase, 11) fumarate hydratase and 12) fumarate reductase.

In addition, we selected two synthetic pathways from an extensive overview of identified synthetic carbon fixation pathways from a study by the Milo group [20]. From these we selected ferredoxin-oxidoreductase containing pathways with both low ATP requirements and favorable kinetics and thermodynamics [20], for which experimental implementation in *E*. *coli* is ongoing [6]: the (v) PyrS-PyrC-Glx and (vi) pyruvate synthase–phosphoenol carboxylase—glyoxylate (PyrS-PEPC-Glx) bicycles (Fig 2). The latter pathway was proposed some decades ago to be a natural cycle, named reductive dicarboxylic acid cycle [35], but its presence in nature could so far not be confirmed [20]. We did not pursue the analysis of the natural 3-hydroxypropionate bicycle–for which engineering has been attempted in *E*. *coli* [7]–because it requires, similar to the natural 3HP-4HB pathway, oxygen or another electron acceptor for the regeneration of the high potential electron acceptor of the succinate dehydrogenase. The natural carbon fixation with the lowest ATP consumption known, the Wood-Ljungdahl pathway [31,32], was also not included, as this complex pathway is probably hard to engineer into *E*. *coli*, as it has complex metallo-chemistry [3].

Carbon uptake was assumed to occur by passive diffusion of CO_2_ over the cell membranes, which is already implemented as a reaction in the applied *E*. *coli* core model. Alternatively, one may consider to increase carbon uptake rate by expressing heterologous bicarbonate transporters. However, such a bicarbonate transporter may not be required in *E*. *coli*. It was demonstrated that heterologous expression of a bicarbonate transporter in *E*. *coli* did not further increase CO_2_ fixation rates in an *E*. *coli* strain engineered with Calvin cycle enzymes [8].

The reactions for all six analyzed carbon fixation pathways are listed in S1 Table. These reactions were used for FBA and FVA in the *E*. *coli* core model and further computational pathway analyses. The carbon dioxide uptake flux was not constrained for FBA and FVA.

#### 2.1.2 Proton-pumping rhodopsin reaction

The PPR photosystem absorbs photons to pump protons from the cytoplasm to the periplasm. However, we use the *E*. *coli* core model for our computational analysis (see Section 2.2.1 and S1 Text), which contains no periplasmic compartment. Due to this we modelled the PPRs by a transport reaction for cytoplasmic protons ($H_{c}^{+}$) to extracellular protons ($H_{e}^{+}$):

$$1\;H_{c}^{+}–>1\;H_{e}^{+}$$

Light or photons were not included as a factor in the model; the constraint of the availability of photons we implicitly incorporated by the constraints we put on the PPR reaction. The range of constraints analyzed for this reaction was based on the range of PPR proton pumping values available from literature: 0–10 mmol H^+^/gCDW/h [11,36]. However, to explore the potential of further optimizing the PPR proton pumping we extended the analyzed range up to 50 mmol H^+^/gCDW/h.

#### 2.1.3. Electron-uptake reactions

To enable carbon fixation, electron donors need to be regenerated continuously. This cannot be done by the proton-pumping rhodopsin photosystems. Hence, those electron donors need to be regenerated through external electron uptake mechanisms. Several external electron donors are promising for electro-autotrophy, e.g. low potential electrons, formate or hydrogen. For practical application, these electron donors basically all require a pathway that couples them to the required electron donors for carbon fixation: NADPH, NADH and/or reduced ferredoxin. Therefore the electron uptake reaction was generalized for FBA and FVA by adding an exchange reaction for extracellular electrons ($e_{e}^{-}$) and subsequent reactions that use these electrons to provide electron donors, similar to the previously published simplified FBA reaction for electrosynthesis [17].

NADPH regeneration:

$$1\;{NADP}^{+}+2\;e_{e}^{-}+1\;H_{c}^{+}–>1\;NADPH$$

We added no reaction to regenerate NADH, since both the *E*. *coli* core and the genome-scale model have an NAD transhydrogenase, which transfers electrons from NADPH to NADH [37].

Ferredoxin_red_ regeneration was implemented in the model as well:

$$1\;{Ferredoxin}_{ox}+1\;e_{e}^{–}–>1\;{Ferredoxin}_{red}$$

It has to be noted, that unlike NAD(P)^+^, the reduction of ferredoxin by H_2_ quickly becomes unfavorable even at moderately low H_2_ concentrations or ferredoxin_red_:ferredoxin_ox_ ratios. However, recently several flavin-based electron bifurcating enzymes [38–40] have been described that couple endergonic reduction of ferredoxin with exergonic reduction of NAD(P)H. Given that the ferredoxin-requiring carbon fixation pathways analyzed, require ferredoxin as well as NAD(P)H, such systems would be suitable for overall energy-neutral, simultaneous regeneration of both types of electron donors. If this option turns out not to be practically feasible, proton motive force-driven enzymes may be employed for ferredoxin reduction, however this would add energetic costs, which are not accounted for in our analyses here.

The uptake of external electron donors and subsequent electron donation reactions were not constrained for FBA and FVA. However, simulated fluxes for optimal growth could be compared to scarce literature data on feasible specific activities for electron uptake mechanisms.

### 2.2. Computational methods of analysis

#### 2.2.1 Flux Balance and Variability Analysis

**Fig 3:**
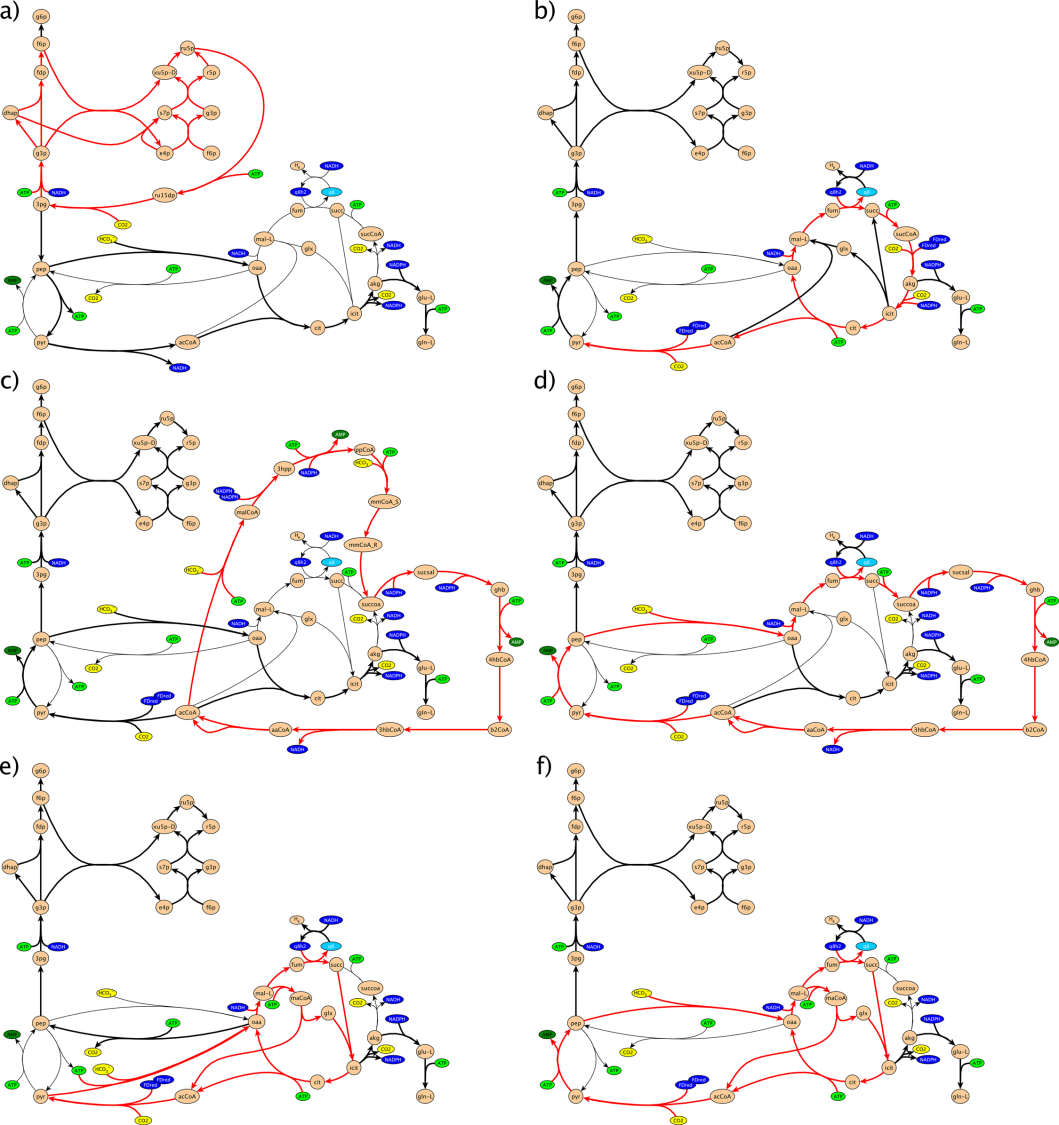
Integration of different carbon fixation pathways in the *E*. *coli* core metabolism. a) Calvin cycle, b) rTCA cycle, c) 3HP-4HB cycle, d) DC-4HB cycle, e) PyrS-PyrC-Glx bicycle, f) PyrS-PEPC-Glx bicycle. Reactions that are part of carbon fixation pathways (red arrows), reactions carrying flux (bold red arrows) and not carrying flux (thin arrows) in the FBA simulations are indicated. ATP (green), electron donors (blue) and CO_2_/HCO_3_^-^ (yellow) are color coded. Reactions of some non-branching pathways were lumped into one reaction for better visualization. For more detailed maps of the carbon fixation pathways see Fig 2 and S1–S4 Figs.

We used the *E*. *coli* core model [41] for FBA [25] and FVA [26] of photo-electro-autotrophic designs in *E*. *coli*. We decided to use the core model instead of the genome-scale model of *E*. *coli*, as it is easier to analyze, for example for assessing total ATP consumption. Furthermore, the core model is more general and not overly specific to *E*. *coli;* this may facilitate more general conclusions that are more likely to apply to other promising chassis organisms having similar central metabolism [30]. Even though all carbon fixation cycles directly link to intermediates in the core metabolism (Fig 3), we verified that the full model gave similar predictions as the core model, to rule out biased results by using the core model (S1 Text and S2 Table). The reactions listed in S1 Table were added to the model, including the missing reactions for the carbon fixation pathways, reactions to provide electron donors and a reaction representing the PPRs. The constraints used for the FBA and FVA are included in S1 and S2 Tables. We performed FBA and FVA with both the standard Non Growth Associated Maintenance (NGAM) for *E*. *coli of* 8.39 mmol ATP/gCDW/h [42] and a lower NGAM estimate of 1.00 mmol ATP/gCDW/h. To check for possible alternative flux distributions for the optimal growth solution, we also performed Flux Variability Analysis (FVA). This is mainly interesting for the calculation of the protein burden to the cell (Section 2.2.5) and the ATP consumption analysis (Section 2.2.2), since in these methods the flux through a certain reaction and the flux distribution, respectively, play a crucial role. For FBA and FVA we used Python 2.7 and the cobrapy toolbox [43] with GLPK 4.48 as the solver for the linear optimization. We optimized for growth utilizing the default biomass objective function in the *E*. *coli* core model [41] (S1 and S2 Tables).

#### 2.2.2 ATP consumption analysis of carbon fixation

We quantified the ATP consumption of the carbon fixation pathways to produce biomass. This was done by calculating the number of ATPs consumed for each precursor in the *E*. *coli* core model biomass equation. All consumed ATPs were calculated, starting from inorganic carbon to the biomass precursors through the pathways taken in the solution from the FBA. We took into account two ATPs consumed for AMP-producing reactions. We calculated the absolute ATP consumption per mole biomass precursor. From this we calculated the total ATP consumption per mole biomass produced, by taking into account the stoichiometry of the precursors in the biomass objective function and the Growth Associated Maintenance (GAM) of the biomass reaction.

#### 2.2.3 Thermodynamic analysis of carbon fixation

For analyzing the thermodynamics of the carbon fixation pathways, Gibbs free energies of reactions (Δ_r_G’^0^) were acquired by the Component Contribution method, implemented in the eQuilibrator software [21,22,24]. The Gibbs free energy of a reaction (Δ_r_G’) under physiological conditions depends on the metabolite concentrations of both substrates and products. These metabolite concentrations can be varied to optimize the thermodynamic profile of the carbon fixation pathways. In this analysis we focus on the reaction’s thermodynamic driving force, that is the negative change of Gibbs free energy (-Δ_r_G’) [23]. A pathway is only thermodynamically feasible if the thermodynamic driving force of each reaction is positive. The thermodynamic profile optimization was done by varying the metabolite concentrations within physiological constraints to maximize the minimal driving force in a pathway. After optimization we found the thermodynamic bottleneck reaction of a pathway, which is the reaction with the minimal thermodynamic driving force. This value for the maximized, minimal thermodynamic driving force of a pathway is further referred to as the Max-min Driving Force (MDF) [23]. This MDF determines the magnitude of the thermodynamic bottleneck of a given pathway, i.e. a higher MDF indicates a less severe thermodynamic bottleneck. The MDF analysis done in here is an improved implementation of the original MDF analysis [23]. This implementation of MDF applied here and elsewhere [44], also incorporates the standard errors in Gibbs free energy estimates made by the Component Contribution method (S2 Text).

For the thermodynamic profile optimization, we fixed some co-factor concentrations and where possible ratios of co-factors, if not stated otherwise. For other metabolite concentrations we chose the range to be between 1 μM and 10 mM, representing a physiological range [23]. The concentration of the two inorganic carbon species consumed in different carbon fixation pathways was fixed: [CO_2_ (aq)] = 0.3 mM, [HCO_3_^-^] = 7 mM. These concentrations are based on pH 7.5 and equilibrium with a gas feed phase containing 1% (v/v) CO_2_, which is a feasible CO_2_ concentration for an industrial set-up. For the following co-factors concentrations and ratios were fixed: [orthophosphate] = 10 mM, [pyrophosphate] = 1 mM, [CoA] = 1 mM, [ATP]/[ADP] = 10, [ADP]/[AMP] = 1, [NADPH]/[NADP^+^] = 10, [NADH]/[NAD^+^] = 0.1 as in [23]. We calculated all the Gibbs free energies for pH 7.5 and an ionic strength of 0.2 M [23]. For [ferredoxin_red_]/[ferredoxin_ox_] we assumed a ratio of 10, contrary to the ratio of 1 assumed before [23]. This ratio can be generally maintained in cells by flavin-based electron bifurcating enzymes as discussed above (Section 2.3.1). Furthermore, we assumed that the PPRs together with the operation of ATP synthase can maintain the [ATP]/[ADP] ratio.

Most concentrations and ratios chosen, are based on the characteristics of *E*. *coli* cytoplasm [23,45]. However, in real life conditions and literature a wide range of co-factor concentrations and ratios can be found. Hence, we also performed a sensitivity analysis for the carbon species concentrations, ratios of electron donor and ATP/ADP. We varied the ratios given above one at a time as follows; [ferredoxin_red_]/[ferredoxin_ox_]: 0.5–50, [ATP]/[ADP]: 5–20, [NADPH]/[NADP^+^]: 0.5–50 and [NADH]/[NAD^+^]: 0.1–10. Additionally, we computed the MDF for a varying range of [CO_2_ (aq)]: 0.01–3 mM and [HCO_3_^-^]: 0.25–70 mM.

We optimized the thermodynamic profile using Python 2.7 and a module for sequential least squares programming from the package scipy.optimize (fmin_slsqp).

#### 2.2.4 Kinetic analysis of carbon fixation pathways

To compare the kinetics of the carbon fixation pathways, we used two methods. The first method, Flux-Force Efficacy (FFE), is related to the thermodynamic driving force of the thermodynamic bottleneck reaction in a pathway, which is quantified by the MDF, as discussed in the section above [23]. The FFE represents the forward flux as a fraction of the total flux through the bottleneck reaction. The FFE has values between 0 and 1; a value close to 1 is preferred as this means that the backward flux through the thermodynamic bottleneck reaction is negligible. The FFE for bottleneck reactions is calculated according to:

$$\text{FFE}=\frac{\text{-}1+\exp(\frac{\text{-}\Delta_{\text{r}}{\text{G}}^{’}}{\text{RT}})}{1+\exp(\frac{\text{-}\Delta_{\text{r}}{\text{G}}^{’}}{\text{RT}})}$$ (1)

*R* is the gas constant (8.314 J/mol/K), *T* is the temperature (310.15 K) and -Δ_r_G’ is the MDF of the pathway.

The second method for estimating kinetics is the Pathway Specific Activity (PSA) [20]. PSA is defined as the upper limit flux (μmol pathway product/mg/min) carried by 1 mg of total protein in the pathway. PSA is calculated according to:

$$\text{PSA}=1/\sum_{\text{i}=1}^{\text{m}}\frac{{\text{w}}_{\text{i}}}{{\text{V}}_{\text{i}}}$$ (2)

With *m*, *w*_*i*_ and *v*_*i*_ as the number of enzymes in the pathway, their stoichiometric coefficients in the pathway and the specific activities of those enzymes, respectively. Most of the specific activities of the enzymes were taken from [20]. The specific activities of some of the reactions in the carbon fixation pathways were not available before, due to a lack of experimental data especially for the ferredoxin-oxidoreductase enzymes [20]. However, recently some new experimental data became available for those enzymes in BRENDA [46]. So, we estimated their specific activity by taking the mean, after discarding the lower 50% and upper 10% of the specific enzyme activities found in BRENDA. These values were discarded to limit the inclusion of unnaturally low or high activities, because of experimental errors and non-representative *in vitro* conditions, as done in [20]. All enzymes and their specific activities are listed in S1 Table. In contrast to [20], we assumed CO_2_ and HCO_3_^-^ concentrations to be saturated, similarly to all other concentrations. This assumption allowed to have the most fair comparison between pathways, since the K_m_ values for the carbon species for the ferredoxin-oxidoreductase enzymes are not available. Furthermore, in a potential industrial application a gas feed with high CO_2_ concentrations close to saturation is feasible. As the pathway end product for PSA we chose pyruvate, since this is a central metabolite, and an important precursor for biosynthesis of many products of commercial interest, further it is an important product of several analyzed carbon fixation pathways.

#### 2.2.5. Protein burden of photo-electro-autotrophic system

We propose the Pathway Protein Burden (PPB) (g protein/gCDW) as a method to estimate the fraction of cellular dry weight that needs to be dedicated to carbon fixation enzymes to produce pyruvate from CO_2_ for the growth rate predicted by FBA. It is defined as:

$$PPB=\frac{F_{{CO}_{2}}}{n*PSA}$$ (3)

where *F*_*CO2*_ (mmol/gCDW/h) is the CO_2_ uptake flux from the FBA for the simulated growth rates, *PSA* is the Pathway Specific Activity (μmol/mg protein/h) and *n* is the number of CO_2_/HCO_3_^-^ molecules needed to produce one molecule of pathway end product. In our analysis this pathway end product is pyruvate, (*n* = 3).

Since PSA is an estimate for the fastest kinetics of a carbon fixation pathway, the PPB represents a minimal estimate for the protein burden of the carbon fixation pathway to support the predicted CO_2_ uptake flux to produce pyruvate.

For electron uptake mechanisms and PPRs, which are mostly single proteins or protein complexes, similarly an estimate for the Protein Burden (PB) (g protein/gCDW) of these systems can be made as follows:

$$PB=\frac{F}{n*SA}$$ (4)

Where *F* (mmol/gCDW/h) is the electron uptake flux or proton-pumping flux for the simulated growth conditions, *SA* (μmol/mg protein/h) is the specific activity of the related protein, i.e. the electron uptake enzyme or PPR. *n* is a stochiometric constant for example for converting the number of electrons per hydrogen or formate molecule (*n* = 2).

## 3. Results and Discussion

### 3.1. FBA and FVA of photo-electro-autotrophic designs

#### 3.1.1 Predicted growth and fluxes depend on carbon fixation cycle

We used FBA to predict photo-electro-autotrophic growth rates for different carbon fixation cycles (Table 2) and to investigate which parts of the central metabolism are possibly used for this growth (Fig 3). Different carbon fixation cycles resulted in different growth rates, which could be related to their ATP requirements, as will be discussed later (Section 3.1.2). Furthermore, we found by FBA that the added reactions for a certain carbon fixation cycle were not always necessarily taken for an optimal growth solution. When we simulated the 3HP-4HB cycle by FBA it only utilized a part of the cycle, i.e. from succinyl-CoA to acetyl-CoA. The subsequent conversion from acetyl-CoA to succinyl-CoA was replaced by a more ATP efficient route involving some of the other core model reactions of *E*. *coli* and the added pyruvate synthase. The cycle taken, turned out to be an alternative natural cycle, the DC-4HB cycle, found in anaerobic archaea [47], which we then included for further analyses (S4 Fig).

**Table 2:** Main *in silico* results for all analyzed carbon fixation pathways. Results shown are based on a high flux of PPR proton pumping of 50 mmol/gCDW/h. We also included the number of enzymes natively present or already heterologously, functionally expressed *in vivo* in *E*. *coli* (S3 Text).

	Flux Balance Analysis (FBA)		Thermodynamics	Stoichiometry	Kinetics		Pathway Protein Burden		Expression challenges		
Carbon fixation pathway	growth rate (hr^-1^)						g protein/gCDW		Number of enzymes		
									non-native in *E*. *coli*		native in *E*. *coli*
	low maintenance (1.00 mmol ATP/gCDW/h)	high maintenance (8.39 mmol ATP/gCDW/h)	Max-Min Driving Force (MDF) (kJ/mol)	ATP required (mmol/gCDW)	Flux-Force Efficacy (FFE)	Pathway Specific Activity (PSA) (μmol pyruvate/mg pathway protein/min)	low maintenance (1.00 mmol ATP/gCDW/h)	high maintenance (8.39 mmol ATP/gCDW/h)	engineered	not engineered	
Calvin cycle	0.083	0.030	4.61	238	0.73	0.86	1.3%	0.5%	2	1	10
rTCA cycle	0.209	0.071	5.66	124	0.82	1.97	1.5%	0.5%	1	1	6
3HP-4HB cycle	0.083	0.030	9.93	227	0.96	0.51	2.3%	0.8%	6	2	5
DC-4HB cycle	0.110	0.039	9.27	203	0.95	0.54	2.8%	1.0%	3	1	10
PyrS-PyrC-Glx bicycle	0.164	0.058	7.11	144	0.89	1.22	1.9%	0,6%	4	0	6
PyrS-PEPC-Glx bicycle	0.130	0.047	7.12	175	0.89	1.10	1.6%	0.6%	3	0	8

Additionally, we analyzed the complete, 3HP-4HB cycle as well, by knocking out succinyl-CoA ligase reaction and thus forcing FBA to use the 3HP-4HB route including pyruvate synthase, which resulted in a lower predicted growth rate than for the DC-4HB cycle. Furthermore, we observed that the FBA carbon fixation simulations resulted in formation of some products, e.g. acetate or formate. These by-products were formed to regenerate some of the ATP used for the carbon fixation, either directly by substrate level phosphorylation or indirectly by generating additional proton motive force via proton symport in the by-product efflux reaction. The by-product formation was driven by additional uptake of electrons, since electron donors were needed to produce those by-products from CO_2_. This led to additional flux for electron uptake, other than for biomass production. In an industrial setting, where photo-electro-autotrophic *E*. *coli* strains would possibly produce a specific product, separating undesired by-products complicates downstream purification of desired products, reducing the economic advantage of the whole process. Hence, we excluded certain reactions to block the generation of all by-products (S1 Table). Whereas this can be easily done in the metabolic model, in an experimental set-up this may require several gene knockouts.

On top of FBA, FVA was performed to analyze if the fluxes and flux distributions found by FBA could vary for the same optimal growth solutions. FVA showed that there is only very little variability in flux distributions to achieve the optimal growth rate given the constraints. Only some futile cycles and side-reactions showed some variability (S2 Table). The overall flux distribution did not vary and, not unexpectedly, the unconstrained uptake rates of CO_2_ and electrons did not vary. The FVA demonstrated that all relevant fluxes which we further apply to estimate ATP consumption and protein burdens gave no variation ranges for the optimal growth scenario, hereby we justified taking the fluxes resulting from FBA for further analysis.

#### 3.1.2 ATP regeneration limits photo-electro-autotrophic growth

We found by FBA that PPR proton-pumping flux and, consequently, ATP regeneration severely limited autotrophic growth rates. We varied the PPR flux between 0 and 50 mmol H^+^/gCDW/h; autotrophic growth was predicted for all carbon fixation pathways only above a threshold PPR flux of 34.0 mmol H^+^/gCDW/h (S5 Fig). At 50 mmol H^+^/gCDW/h growths rates varied from 0.030 h^-1^ (Calvin cycle and 3HP-4HB cycle) to 0.071 h^-1^ (rTCA cycle). This PPR flux threshold is caused by the fact that the non-growth associated ATP requirement for maintenance (NGAM) needs to be overcome before ATP is available for growth. *E*. *coli* normally has a NGAM flux of 8.39 mmol ATP/gCDW/h. This NGAM value is based on standard heterotrophic growth conditions and includes ATP consumption for other processes than anabolic reactions, e.g. membrane leakage [42].

The NGAM value is normally determined in chemostat experiments. However, for a completely new growth phenotype of an engineered, and potentially laboratory evolved, autotrophic *E*. *coli*, a reliable estimate for the maintenance term is not available and it may turn out to be higher or lower [30]. The burden of heterologous expression of autotrophic components may increase the NGAM, which would make the designed photo-electro-autotrophic designs infeasible. Therefore proper estimates of the ATP burden of heterologous expression [48], of for example for autotrophy pathways, and methods to minimize this burden are of key importance. On the other hand, nature demonstrates that lower NGAM values than the standard *E*. *coli* value are feasible, as for several organisms lower NGAM values have been determined. For example, for the strictly anearobic *Shewanella oneidensis* and *Geobacter metallireducens*, which both can transfer external electrons directly, NGAM values were determined to be 1.03 and 0.81 mmol ATP/gCDW/h, respectively [49,50]. The anoxygenic photosynthetic *Rhodobacter sphaeroides* has a NGAM of 8.4 ATP/gCDW/h during photoheterotrophic growth at high light intensities, but during light-limited photoheterotrophic growth this can decrease down to 0.5 ATP/gCDW/h [51].

Based on this naturally feasible lower range of NGAM values, we repeated our FBA simulations with a lower NGAM of 1.00 mmol ATP/gCWD/h. With the low NGAM, the PPR flux threshold above which growth is feasible decreased to 4.0 mmol H^+^/gCDW/h (S6 Fig). Consequently, the maximum growth rates at a PPR flux of 50 mmol H^+^/gCDW/h increased substantially compared to the high NGAM scenario (Table 2). These maximum growth rates varied from 0.083 h^-1^ (Calvin cycle and 3HP-4HB cycle) to 0.209 h^-1^ (rTCA cycle). However, when we considered a somewhat more realistic proton flux for the PPRs of 20.0 mmol H^+^/gCDW/h, for the ATP-efficient synthetic PyrS-PyrC-Glx bicycle, a growth rate of 0.057 h^-1^ seemed feasible. Such a growth rate would be a high growth rate for autotrophs, considering that most natural autotrophic organisms have growth rates below 0.1 h^-1^. For instance, the photosynthetic model cyanobacterium *Synechocystis* PCC6803 has a maximum photoautotrophic growth rate of 0.085 h^-1^ [52,53]. However, a photo-electro-autotrophic *E*. *coli* may only achieve growth rates in this range if several criteria are met: a low NGAM, a high PPR flux and an ATP-efficient CO_2_ fixation cycle.

As PPR proton export was generally limiting growth, we analyzed proton-pumping reactions in the FBA in more detail. The PPR mainly created the proton efflux from the cytoplasm. Furthermore, the pathways that included fumarate reductase (rTCA, DC-4HB, PyrS-PyrC-Glx and PyrS-PEPC-Glx) had an additional efflux of protons from ubiquinol recovery (S7 Fig). For most organisms harboring the rTCA, the natural electron donor for fumarate reductase is not known, though in *Hydrogenobacter* it is reported to be NADH [31]. Alternatively, in *E*. *coli* and consequently in our analyses, a native ubiquinol-dependent fumarate reductase could be employed for the rTCA cycle, DC-HB cycle and the synthetic bicycles. The reduction of resulting ubiquinone via an NADH dehydrogenase created an additional export of protons. The majority of available proton motive force resulting from proton efflux, was imported by the ATP synthase to regenerate ATP. The influx through the ATP synthase was even larger than the total efflux of PPRs and ubiquinol-dependent reductases. This gap could be explained by additional proton uptake from the external medium, which we could relate to the formation of water in the carbon fixation pathways (S7 Fig).

In addition to the PPR proton export flux we analyzed external electron uptake flux and CO_2_ uptake flux for all scenarios. The maximum electron uptake varied from 14.4 mmol e^-^/gCDW/h for the cycles with the slowest predicted growth (3HP-4HB and Calvin cycle) to 35.7 mmol e^-^/gCDW/h for the fastest predicted growth (rTCA cycle) (S8 Fig). Accordingly, the CO_2_ uptake ranged from 3.5 mmol CO_2_/gCDW/h (both, 3HP-4HB and Calvin cycle)) to 8.7 mmol CO_2_/gCDW/h (rTCA cycle) (S8 Fig). The feasibility of all these fluxes is related to the kinetic activities of related enzymes and the resulting protein burdens, which is discussed in section 3.5. Summarizing the electron and CO_2_ uptake flux practically limit photo-electro-autotrophic growth to a lesser extent than the PPR proton-pumping flux.

### 3.2. ATP consumption of carbon fixation pathways

By FBA we found that the PPR flux, and consequently the ATP production, was the limiting factor for the photo-electro-autotrophic growth of *E*. *coli*. In addition, we found considerably varying growth rates for the different carbon fixation pathways. Therefore we analyzed in more detail the ATP consumption per mole biomass formed for every carbon fixation pathway, given the reactions toward biomass precursors that carry flux according to FBA. The FVA showed that there is no variability in the reactions toward biomass precursors that carry flux, which justifies using the results from FBA as a basis for this ATP calculation.

**Fig 4:**
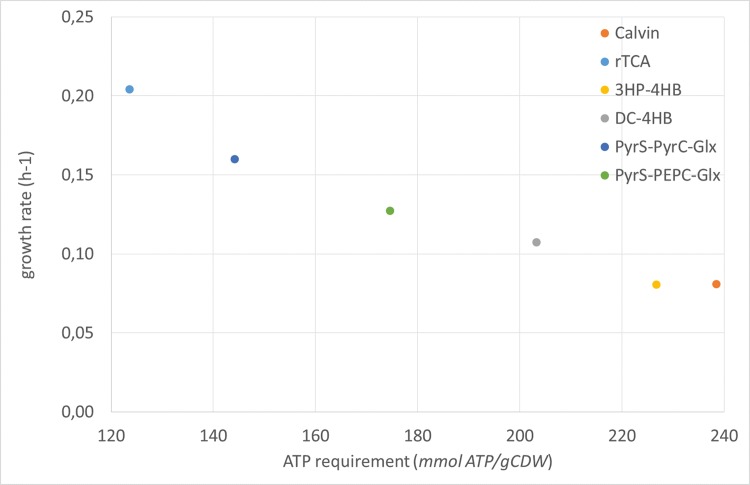
Photo-electro-autotrophic growth rates in relation to the ATP requirement for biomass. Growth rates were based on a PPR flux of 50.0 mmol H^+^/gCDW/h and a NGAM of 1.00 mmol ATP/gCDW/h.

The ATP consumption per mole biomass consists of the ATP needed for the biosynthesis of biomass precursors and the Growth Associated Maintenance (GAM). For *E*. *coli* this GAM is 59.8 mmol ATP/gCDW, independent of the carbon fixation pathway utilized, whereas the ATP requirement for the biomass precursors is different for each carbon fixation pathway. Therefore, the total ATP requirement varied from 124 mmol ATP/gCDW (rTCA cycle) to 238 mmol ATP/gCDW (Calvin cycle) (Table 2, S9 Fig). The ATP requirement per mole biomass is negatively correlated with the growth rates predicted by the FBA for the pathways (Fig 4). Roughly the rTCA cycle and the PyrS-PyrC-Glx bicycle had the lowest ATP requirements and gave clearly higher growth rates in the FBA than the other cycles. The 3HP-4HB cycle had a growth rate similar to the Calvin cycle, even though the ATP requirement is lower for the 3HP-4HB cycle (227 mmol ATP/gCDW). This is related to the fact that the 3HP-4HB cycle generates NADH (S3 Fig) which needs to be regenerated to NADPH by consuming some of the exported protons (S7 Fig).

### 3.3. Thermodynamics of carbon fixation under different conditions

We determined MDF values for all carbon fixation pathways to determine their thermodynamical favorability. A negative MDF means the pathway is not feasible, and a higher MDF value indicates that a pathway is thermodynamically more favorable (Section 2.2.3 and [23]). Our analysis showed that under standard conditions all pathways were feasible. The natural 3-HP-4HB cycle and DC-4HB cycle were thermodynamically most favorable. Also both synthetic bicycles were very favorable, the Calvin cycle and rTCA cycle were slightly less favorable (Table 2).

To check how sensitive the thermodynamics were if the conditions differed from the defined standard conditions we varied the pH, as well as important co-factor concentrations and ratios. From this, we observed that for a pH range between 5.0 and 9.0 all pathways were thermodynamically feasible (S10 Fig). The DC-4HB and 3HP-4HB cycles had a very low MDF for pH values close to 5.0, however, this pH is already far off from the physiological range of *E*. *coli* (pH ~7.5). So thermodynamically these pathways seemed all quite robust for pH deviations around the *E*. *coli* optimum.

With respect to the co-factors, the sensitivity analysis showed that the ferredoxin-utilizing pathways were thermodynamically less favorable (MDF < 4.0 kJ/mol) when the ferredoxin_red_/ferredoxin_ox_ ratio got very low, i.e. 0.5 (S11 Fig), in particular for the rTCA cycle. This would practically not be a problem if there is an electron uptake mechanism and sufficient external electron donors, thereby maintaining a sufficiently high ratio. However, as discussed in Section 2.3.1., this low-potential redox couple will require systems, such as flavin-based bifurcating enzymes to allow for the thermodynamically less favorable reduction of ferredoxin_ox._.

Low NADPH/NADPH^+^ ratios had only a limited effect on thermodynamic favorability, however again the rTCA had the strongest decrease in MDF values when this cofactor ratio dropped (S12 Fig). A low NADH/NAD^+^ ratio had only a large impact of thermodynamic favorability of the Calvin cycle, the sole electron donor in this cycle, at the typical physiological ratio of 0.1 or lower the MDF dropped below 4 kJ/mol (S13 Fig). It has to be noted that in natural organisms harboring the Calvin cycle, instead of NADH, NADPH is employed as the electron donor. However in our analysis we primarily included NADH as electron donor, as *E*. *coli* natively harbors a reversible glyceraldehyde-3-phosphate dehydrogenase that uses NADH. Given the much higher typical ratio of NADPH:NADP (10), substituting this enzyme by a heterologous variant of using NADPH as electron donor would result in a higher MDF value of around 6 kJ/mol (S13 Fig), as was found in the sensitivity analysis for the physiologically unrealistic NADH:NAD ratio of 10. So to improve thermodynamic favorability of the Calvin cycle the electron donor specificity of glyceraldehyde-3phosphate dehydrogenase is crucial.

The ATP/ADP ratio only had a small impact on thermodynamic favorability of all pathways (S14 Fig). Another thermodynamically critical factor could be the dissolved CO_2_ concentration range. However, even at a dissolved CO_2_ concentration of 10 μM all cycles were still feasible, which would be the dissolved concentration range resulting from an equilibrium with ambient air CO_2_ (S15 Fig). The Calvin cycle was thermodynamically not sensitive to dissolved CO_2_ concentration changes; for all other cycles the MDF dropped to lower, but still positive values for lower dissolved CO_2_. Especially the rTCA got a relatively low thermodynamic favorability at the lowest CO_2_ concentration. HCO_3_^-^ concentrations did not impact the thermodynamic bottleneck reactions for any of the cycles (S16 Fig).

Summarizing, the rTCA cycle and Calvin cycle were generally slightly less thermodynamically favorable than the other analyzed natural and synthetic cycles. Especially at less favorable ratios of electron donors these cycles had lower thermodynamic favorability for their bottleneck reactions, which may result in lower net fluxes through these reactions.

### 3.4. Kinetics of carbon fixation pathways

For analyzing the kinetics of the carbon fixation pathways we calculated both the FFE and PSA (Table 2). The 3-HP-4HB cycle, DC-4HB cycle and the synthetic bicycles all had a FFE of 0.89 or higher, which is related to their higher thermodynamic favorability as discussed above. The rTCA cycle and Calvin cycle therefore had a slightly lower FFE value (Table 2), however still the majority of the flux of the thermodynamic bottleneck reactions was in the forward direction. The highest PSA value was found for the rTCA cycle (1.97 μmol pyruvate/mg pathway protein/min), partly because this cycle involves the lowest number of enzymes. Both synthetic bicycles have the also relatively high with PSA values around 1.1 μmol pyruvate/mg pathway protein/min. The other three natural cycles had the slowest kinetics calculated by PSA. In contrast to an earlier PSA analysis [20], now all PSA values could be compared among the different pathways. As more specific activities for the ferredoxin:oxidoreductase enzymes were available now and included in our analysis [46]. In summary, the synthetic PyrS-PyrC-Glx, PyrS-PEPC-Glx bicycles presented the best combination of FFE and PSA scores, whereas the rTCA cycle had a notably high PSA but a somewhat lower FFE due to its lower thermodynamic favorability. This makes the synthetic bicycles most promising from a pathway kinetics point of view.

### 3.5. Protein burden of photo-electro-autotrophic systems

#### 3.5.1. Carbon fixation pathways

To determine the protein burden of each carbon fixation pathway, we calculated the Pathway Protein Burden (PP) (Section 2.2.5 and Table 2). For the higher NGAM scenario lower protein burdens for carbon fixation were found than for the low NGAM scenario; as for a high NGAM scenario lower growth rates were predicted, consequently lower carbon fixation fluxes and related pathway proteins were required. By assuming a very low NGAM and a high PPR flux, the more promising growth rates were found, in this scenario the smallest protein burden was found for the Calvin cycle (1.3%). This is surprising, as the carboxylase of the Calvin cycle, RubisCO (ribulose-bisphosphate carboxylase-oxygenase) is a notoriously slow catalyst, which therefore is expressed highly in many natural organisms[20]. This kinetic inefficiency of RubisCO is also reflected in the relatively high PSA for the Calvin cycle, however given the low growth rate feasible for this pathway, the overall protein burden for CO_2_ fixation is still the lowest. Other than for the Calvin cycle, low burdens were found for the cycles that resulted in fastest growth,: rTCA cycle (1.5%), the PyrS-PEPC-Glx bicycle (1.6%) and the PyrS-PyrC-Glx bicycle (1.9%). For these cycles the extra pathway protein requirement to sustain their higher CO_2_ fixation flux was compensated by their high PSA values. The other nature cycles had a higher burden up to 2.8% for the DC-4HB cycle.

Under the assumption that the total proteome of cells is about 50% of the cell dry weight [30], the burden of the carbon fixation pathways with a PPB of ~2% of the cell dry weight resulted in a proteome burden of 4% of total cell protein weight. A fraction of 4% heterologous protein expression is well below the 20% considered to be a limit beyond which cell viability is critically affected [30]. However, for a photo-electro-autotrophic *E*. *coli* additional heterologous proteins need to be expressed for PPRs and electron uptake enzymes, as will be discussed below.

#### 3.5.2. Proton-pumping rhodopsin systems

The expression of proton-pumping rhodopsin systems (PPRs) imposes a significant additional protein burden to the cell. For the PPRs based on literature, a proton-pumping activity of 300 mmol H^+^/mmol PPR/min may be feasible [11,36]. Assuming a molecular weight for proteorhodopsin of 29 kDa [54], and hence a specific activity of 10.3 μmol H^+^/mg protein/min, this would give a substantial protein burden of 8.1% at the maximum pumping rate (50 mmol H^+^/gCDW/h) used in FBA. Assuming this specific activity and 3*10^−13^ gCDW/cell, one cell would require to have ~500,000 PPRs/cell, while so far only levels up to 100,000/cell have been measured [11]. A protein burden of 8.1% per CDW corresponds to ~ 16% of the total proteome, and as the cytoplasmic membrane contains about 20–30% of the total proteome [55],so this is a very significant burden to the membrane proteome. So to achieve a high growth by PPRs, either the pumping rates or the number of PPRs/cell has to be significantly increased. Increased membrane surface area may be required to accommodate a higher number of PPRs, analogous to the extra thylakoid membranes in which cyanobacteria accommodate their photosystems.

#### 3.5.3. Electron uptake mechanisms

For estimating the protein burdens of electron uptake mechanisms specific activities of the relevant enzymes are needed. With formate as an electron donor, formate dehydrogenase is needed, for this enzyme some specific activity values are available [56]. For the aerobic, engineered NADPH-dependent formate dehydrogenases the highest specific activity reported is 2.5 μmol formate/mg protein/min [56]. At the maximum electron uptake predicted by FBA this would give an infeasibly high protein burden of 23.8% of cell dry weight. However, for simple aerobic NADH-dependent formate dehydrogenases values up to 10 μmol formate/mg protein/min have been reported, reducing their burden fourfold to 6.0% of cell dry weight. This would still result in a significant burden on the proteome of ~12%. However, for the anaerobic scenario oxygen-sensitive formate dehydrogenases may even allow for higher specific activities and lower burdens[57], but not many reliable values are available yet.

For using hydrogen as an electron donor, hydrogenases are required that are NADP^+^-dependent, ferredoxin-dependent and/or bifurcating. For those no reliable specific activities with physiological electron acceptors were measured yet. Therefore, the protein burden for hydrogen uptake systems, including flavin-based bifurcating enzymes, could not be estimated. Also for direct electron uptake mechanisms also very limited kinetic data are known, however before it was assumed that an electron uptake flux of 30.0 mmol e^-^/gCDW/h is feasible in engineered *E*. *coli* [17], this is close to the maximum flux of 35.7 mmol e^-^/gCDW/h that is required for our highest growth scenario (low NGAM with rTCA cycle).

The estimates here for protein burdens are all based on kinetic data from *in vitro* assays, which not necessarily correspond directly to *in vivo* conditions, but it was recently demonstrated that *in vitro* activities generally concur with *in vivo* activities [58]. Based on burden estimates, for fast photo-electro-autotrophic growth assuming a high proton-pumping rate and low NGAM a total burden on the proteome of at least 20% is estimated to be put on the organism, consisting of a burden of PPRs (~16%) and a burden of the CO_2_ fixation cycle (e.g. ~4% for synthetic bicycles and rTCA). This still excludes the burden for electron uptake, which may be high as well, but is hard to estimate given limited data. To reduce the added burden of this electron uptake, kinetically fast uptake enzymes have to be found or engineered. In comparison, in the photosynthetic cyanobacterium *Synechocystis* sp. PCC6803, 26% of the proteome mass is devoted to energy metabolism [59,60]. The majority is also for the photosystem (18%) and a smaller part for carbon fixation (2.9%). So generally the photosystems seem to give a relative large contribution to the proteome of a photoautotroph.

### 3.6. Challenges for experimental implementation of photo-electro-autotrophic systems

The analysis of the presented designs suggests that photo-electro-autotrophic growth of *E*. *coli* has a limited feasibility. In general, ATP limited the growth rate and the available models cannot predict what the effective ATP maintenance will be for an engineered photo-electro-autotrophic *E*. *coli*. Better *in silico* prediction of NGAM and strategies for minimizing the maintenance burden in engineered cells requires a lot of attention.

For experimentally implementing anaerobic photo-electro-autotrophy the limitation of growth by ATP, related to the PPR proton export flux is a key challenge. It is therefore crucial to find ways to increase the kinetic rates of the proton-pumping rhodopsin photosystems and/or to reach higher numbers of PPRs per cell without surpassing heterologous expression limits in the membrane. In a previous bioenergetic analysis of PPRs in natural photoheterotrophs it was also shown that PPRs generate a relatively low amount of metabolic energy at relatively high protein costs [61], also confirmed experimentally by only very small growth advantages measured for microorganisms heterologously expressing rhodopsins [12,62]. Therefore, research efforts could be aim to increase their energetic efficiency, e.g. by broadening the PPR light absorption and/or proton-pumping kinetics, e.g. by applying PPRs with an antennae pigment [11,63] or through protein engineering of PPRs [64]. If PPRs turn out not to be able to generate sufficient proton pumping for ATP regeneration, it could be considered (first *in silico*) to heterologously express other, more complex (anaerobic) photosystems [65]. One could potentially apply the photosystems found anoxygenic photosynthetic organisms, which are natural photo-electro-autotrophs. These microorganisms generate ATP by their anoxygenic photosystems and use sulfur, hydrogen sulfide or hydrogen as an electron source [66]. Alternatively, these anoxygenic photosynthetic microorganisms themselves may be employed as chassis for engineering photo-electro-autotrophic production. Some basic tools for genetic and metabolic engineering are available for some of these organisms, such as for the green bacterium *Chlorobaculum tepidum*[67] and purple bacterium *Rhodobacter sphaeroides* [68,69].

Apart from applying more efficient photosystems for ATP regeneration, electron acceptors could be used to allow for oxidative phosphorylation, such as oxygen or nitrate. However, oxygen may inhibit certain electron uptake pathway enzymes and carbon fixation pathway enzymes, but there are indications that for some of the oxygen-sensitive enzymes there are natural, oxygen-tolerant alternatives [31]. This could facilitate a (micro)aerobic scenario for engineering electro-autotrophy, where instead of photophosphorylation by PPRs, oxidative phosphorylation is used for ATP production. In such a (micro)aerobic scenario, the favorable and ATP-efficient synthetic carbon fixation pathways can still be advantageous compared to other less ATP-efficient (aerobic) pathways for potentially high product and biomass yields. However, this scenario is only advantageous if the required oxygen-protecting mechanisms of oxygen-tolerant enzymes are not too energy-demanding. Alternatively, for such (micro)aerobic conditions other synthetic non-ferredoxin-dependent, ATP-efficient pathways could be promising and should be analyzed [20].

This work also has shown an extensive integrated analysis of several CO_2_ fixation pathways. These results may guide choices for engineering CO_2_ fixations cycles in autotrophs in general. Given their relatively low ATP costs, especially the rTCA cycle and synthetic PyrS-PyrC-Glx seem to be attractive, resulting in the highest predicted growth for anaerobic photo-electro-autotrophy. In addition to their ATP efficiency, these pathways were found to have a high kinetic activity, resulting in a limited protein burden. The synthetic PyrS-PyrC-glx cycle, may despite its slightly higher ATP consumption and lower growth rates, still be advantageous compared to the rTCA if the less favorable thermodynamics of the rTCA cycle are taken into account.

Furthermore, experimental implementation of these carbon fixation pathways in *E*. *coli* requires functional expression of the pathway enzymes. Therefore, we reviewed native presence of all carbon fixation pathway enzymes in *E*. *coli*, and if absent, we checked literature for reports of successful heterologous expression in *E*. *coli* (S3 Text). Accordingly, we determined the number of native *E*. *coli* enzymes and the number of already heterologously expressed, *in vivo* functionally enzymes (Table 2). The analysis shows that for the synthetic PyrS-PyrC-Glx bicycle only 4 non-native enzymes are needed. All of them have already been (individually) expressed heterologously in *E*. *coli* recently [6]. For the rTCA cycle only 2 enzymes have to be heterologously expressed, however running the reverse TCA cycle in *E*. *coli* will probably also involve reregulation of the native oxidative TCA cycle. As the PyrS-PyrC-Glx bicycle and the rTCA cycle require reduced ferredoxin, attention has to be paid to achieve heterologous expression of e.g. recently discovered flavin-based bifurcating enzymes in *E*. *coli*, which allow for thermodynamically favorable ferredoxin reduction.

Also other hosts than *E*. *coli* may be considered for engineering (photo-electro-)autotrophy, as *E*. *coli* for example may appear to have high ATP maintenance costs. A more detailed analysis of the potential of (photo-electro-)autotrophic designs in other hosts, as performed in this work, can be performed if a core or genome-scale metabolic model is available for that host. Promising alternative chassis organisms could be acetogens or methanogens, some of which are genetically accessible [70,71]. Acetogens and methanogens harbor the extremely ATP-efficient Wood-Ljungdahl carbon fixation pathway and they generally have uptake mechanisms for hydrogen. However, these organisms produce large amounts of acetate or methane to generate ATP by substrate level phosphorylation; these are mostly unwanted by-products for industrial applications. This by-product formation could potentially be avoided by introduction of PPRs for photophosphorylation into these organisms. This could be analyzed *in silico* using the approach described in this paper.

In summary, the herein described integrated *in silico* analysis is an in-depth exploration of the potential of photo-electro-autotrophy to indicate bottlenecks and steer strategic choices before experimental implementation. We envision that such an integration of tools will prove to be very useful in developing and analyzing designs for synthetic autotrophy and other challenging pathway designs in synthetic biology.

## Supporting Information

S1 Fig Calvin cycle. Reactions are: 1) Triose-phosphate isomerase, 2) Fructose-bisphosphate aldolase, 3) Fructose-bisphosphatase, 4) Transketolase, 5) Sedoheptulose-1,7-bisphosphate/fructose-1,7-bisphosphate aldolase, 6) Sedoheptulose-bisphosphatase, 7) Transketolase, 8) Ribose-5-phosphate isomerase, 9) Ribulose-phosphate 3-epimerase, 10) Phosphoribulokinase, 11) Ribulose-bisphosphate carboxylase, 12) Phosphoglycerate kinase and 13) Glyceraldehyde-3-phosphate dehydrogenase (phosphorylating).
(EPS)

S2 Fig rTCA cycle. Reactions are: 1) Malate dehydrogenase, 2) Fumarate hydratase, 3) fumarate reductase, 4) Succinate-CoA ligase, 5) 2-oxoglutarate:ferredoxin-oxidoreductase synthase, 6) Isocitrate dehydrogenase, 7) Aconitate hydratase and 8) ATP citrate lyase.
(EPS)

S3 Fig 3HP–4HB cycle. Reactions are: 1) Acetyl-CoA carboxylase, 2) Malonyl-CoA reductase (bifunctional enzyme), 3) Propionyl-CoA synthase (tri-functional enzyme), 4) Propionyl-CoA carboxylase, 5) Methylmalonyl-CoA epimerase, 6) Methylmalonyl-CoA mutase, 7) Succinyl-CoA reductase, 8) Succinate-semialdehyde reductase, 9) 4-hydroxybutyryl-CoA synthetase, 10) 4-hydroxybutyryl-CoA dehydratase, 11) 3-hydroxyacyl-CoA dehydratase (3-hydroxybutanoyl-CoA), 12) 3-hydroxyacyl-CoA dehydrogenase (acetoacetyl-CoA) and 13) Acetyl-CoA C-acetyltransferase.
(EPS)

S4 Fig DC-4HB cycle. Reactions are: 1) Pyruvate synthase, 2) Pyruvate water dikinase, 3) Phosphoenolpyruvate carboxylase, 4) Malate dehydrogenase, 5) Fumarate hydratase, 6) fumarate reductase, 7) Succinate-CoA ligase, 8) Succinyl-CoA reductase, 9) Succinate-semialdehyde reductase, 10) 4-hydroxybutyryl-CoA synthetase, 11) 4-hydroxybutyryl-CoA dehydratase, 12) 3-hydroxyacyl-CoA dehydratase (3-hydroxybutanoyl-CoA), 13) 3-hydroxyacyl-CoA dehydrogenase (acetoacetyl-CoA) and 14) Acetyl-CoA C-acetyltransferase.
(EPS)

S5 Fig Growth rates for photo-electro-autotroph *E*. *coli* with high maintenance. Predicted by FBA for different carbon fixation pathways with a NGAM of 8.39 mmol ATP/gCDW/h. PPR proton flux was varied between 0 and 50 mmol H^+^/gCDW/h.
(TIF)

S6 Fig Growth rates for photo-electro-autotroph *E*. *coli* with low maintenance. Predicted by FBA for different carbon fixation pathways with a NGAM of 1.00 mmol ATP/gCDW/h. PPR proton flux was varied between 0 and 50 mmol H^+^/gCDW/h.
(TIF)

S7 Fig Proton fluxes related to proton import through ATP-synthase. Major proton fluxes in and out of the cell during at a proton-pumping flux of 50 mmol H^+^/gCDW/h and a NGAM of 1.00 mmol ATP/gCDW/h
(TIF)

S8 Fig Electron and CO_2_ uptake predicted by FBA for different carbon_2_ fixation pathways with a NGAM of 1.00 mmol ATP/gCDW/h. Proton pumping was varied between 0 and 50 mmol H^+^/gCDW/h.
(TIF)

S9 Fig ATP requirements for the biomass precursors of the *E*. *coli* core model for different carbon fixation pathways. Biomass precursors are: D-glucose-6-phosphate (g6p), D-fructose-6-phosphate (f6p), D-erythrose-4-phosphate (e4p), *α*-D-ribose-5-phosphate (r5p), glyceraldehyde-3-phosphate (g3p), 3-phospho-D-glycerate (3pg), phosphoenolpyruvate (pep), pyruvate (pyr), acetyl-CoA (accoa), oxaloacetate (oaa), L-glutamate (glu_L) and L-glutamine (gln_L).
(TIF)

S10 Fig Max-Min Driving Force (MDF) for varying pH values. (TIF)

S11 Fig Max-Min Driving Force (MDF) for varying ratios of reduced ferredoxin and oxidized ferredoxin (FD_red_/FD_ox_). Bar labels indicate which reaction results in this MDF values, so which reaction forms the thermodynamic bottleneck. Bottleneck reactions are: glyceraldehyde-3-phosphate dehydrogenase (GAPD), isocitrate dehydrogenase (ICDHyr), 2-oxoglutarate:ferredoxin-oxidoreductase synthase (OGOR), succinyl-CoA synthetase (SUCOAS), pyruvate synthase (PYRS), (S)-malyl-CoA lyase (MCL), 3-hydroxyacyl-CoA dehydrogenase (HACD1), 3-hydroxyacyl-CoA dehydratase (ECOAH1).
(TIF)

S12 Fig Max-Min Driving Force (MDF) for varying ratios of NADPH/NADP^+^. Bar labels indicate which reaction results in this MDF values, so which reaction forms the thermodynamic bottleneck. Bottleneck reactions are: glyceraldehyde-3-phosphate dehydrogenase (GAPD), isocitrate dehydrogenase (ICDHyr), succinyl-CoA synthetase (SUCOAS), pyruvate synthase (PYRS), 3-hydroxyacyl-CoA dehydrogenase (HACD1), acetyl-CoA C-acetyltransferase (ACACT1r).
(TIF)

S13 Fig Max-Min Driving Force (MDF) for varying ratios of NADH/NAD^+^. Bar labels indicate which reaction results in this MDF values, so which reaction forms the thermodynamic bottleneck. Bottleneck reactions are: glyceraldehyde-3-phosphate dehydrogenase (GAPD), ribose-5-phosphate isomerase (RPI), transketolase (TKT2), isocitrate dehydrogenase (ICDHyr), pyruvate synthase (PYRS), pyruvate carboxylase (PYRC), ATP citrate lyase (ACL), 3-hydroxyacyl-CoA dehydrogenase (HACD1), 3-hydroxyacyl-CoA dehydratase (ECOAH1).
(TIF)

S14 Fig Max-Min Driving Force (MDF) for varying ratios of ATP/ADP. Bar labels indicate which reaction results in this MDF values, so which reaction forms the thermodynamic bottleneck. Bottleneck reactions are: ribose-5-phosphate isomerase (RPI), glyceraldehyde-3-phosphate dehydrogenase (GAPD), succinyl-CoA synthetase (SUCOAS), isocitrate dehydrogenase (ICDHyr), malate dehydrogenase (MDH), pyruvate synthase (PYRS), malyl-CoA synthetase (MTK), 3-hydroxyacyl-CoA dehydrogenase (HACD1), 4-hydroxybutyryl-CoA dehydratase (HB4COADH).
(TIF)

S15 Fig Max-Min Driving Force (MDF) for varying CO_2_ concentrations (M). Bar labels indicate which reaction results in this MDF values, so which reaction forms the thermodynamic bottleneck. Bottleneck reactions are: glyceraldehyde-3-phosphate dehydrogenase (GAPD), ribulose-5-phosphate 3-epimerase (RPE), isocitrate dehydrogenase (ICDHyr), pyruvate synthase (PYRS), malate dehydrogenase (MDH), citrate hydrolyase (CITHL), 3-hydroxyacyl-CoA dehydrogenase (HACD1), 3-hydroxyacyl-CoA dehydratase (ECOAH1).
(TIF)

S16 Fig Max-Min Driving Force (MDF) for varying HCO_3_^-^ concentrations (M). Bar labels indicate which reaction results in this MDF values, so which reaction forms the thermodynamic bottleneck. Bottleneck reactions are: glyceraldehyde-3-phosphate dehydrogenase (GAPD), isocitrate dehydrogenase (ICDHyr), malate dehydrogenase (MDH), (S)-malyl-CoA lyase (MCL), pyruvate synthase (PYRS), 3-hydroxyacyl-CoA dehydratase (ECOAH1), 3-hydroxyacyl-CoA dehydrogenase (HACD1).
(TIF)

S1 Table Reactions and related data used for FBA, MDF and kinetic analysis. (XLSX)

S2 Table Reactions, constraints and results of FBA and FVA. (XLSX)

S1 Text Comparison of FBA results of core model versus genome-scale model. (PDF)

S2 Text Max-min driving force (update). (PDF)

S3 Text Presence and heterologous expression of carbon fixation enzymes in *E*. *coli*. (PDF)
