# Supplementary figures and images for: Integrated In Silico Analysis of Pathway Designs for Synthetic Photo-Electro-Autotrophy
Source: PLoS One. 2016 Jun 23;11(6):e0157851. doi: 10.1371/journal.pone.0157851 (PMC4919048; doi:10.1371/journal.pone.0157851)

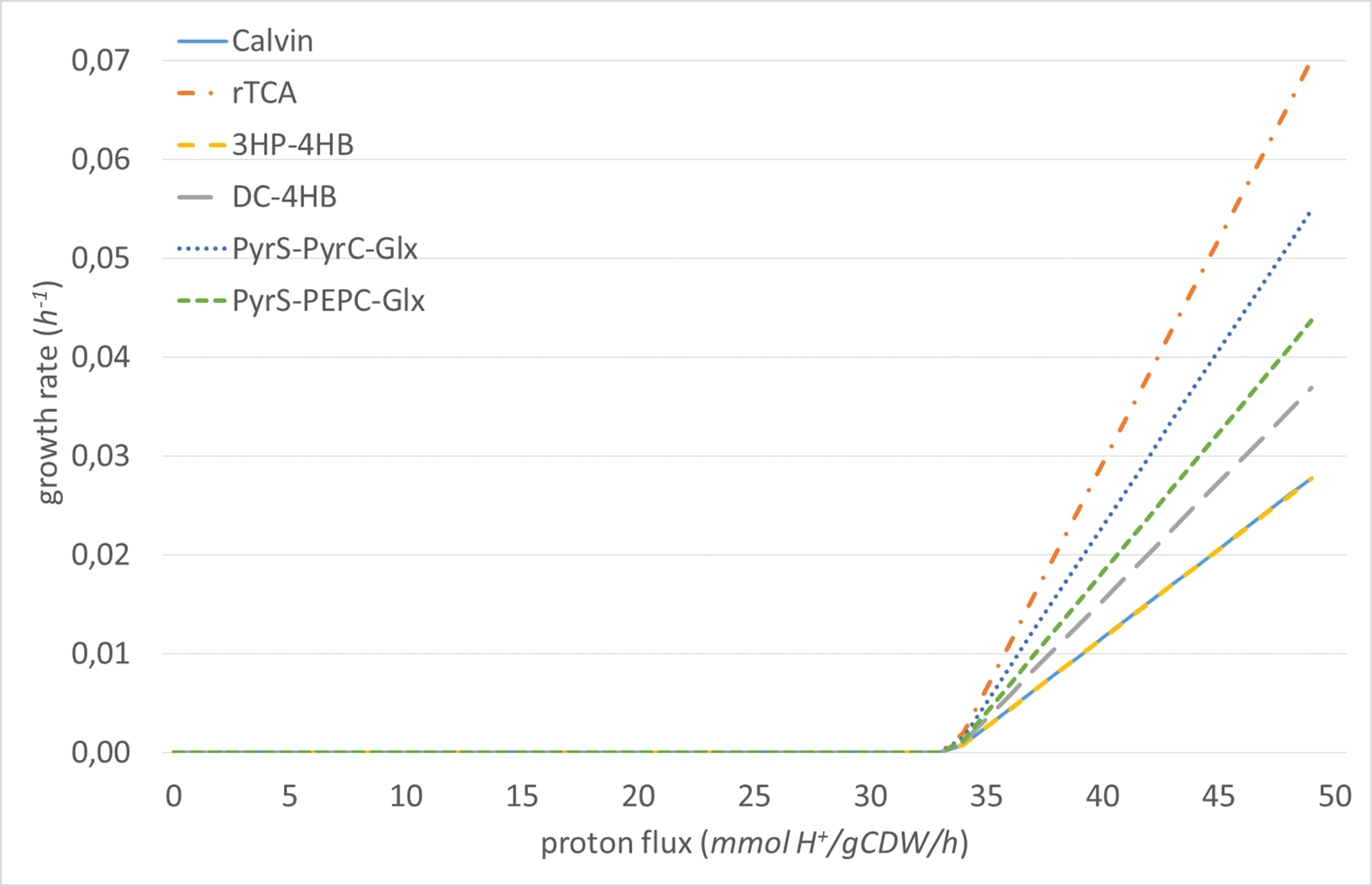

Supplement: S5 Fig — Predicted by FBA for different carbon fixation pathways with a NGAM of 8.39 mmol ATP/gCDW/h. PPR proton flux was varied between 0 and 50 mmol H+/gCDW/h. (TIF) [file pone.0157851.s005.tif]

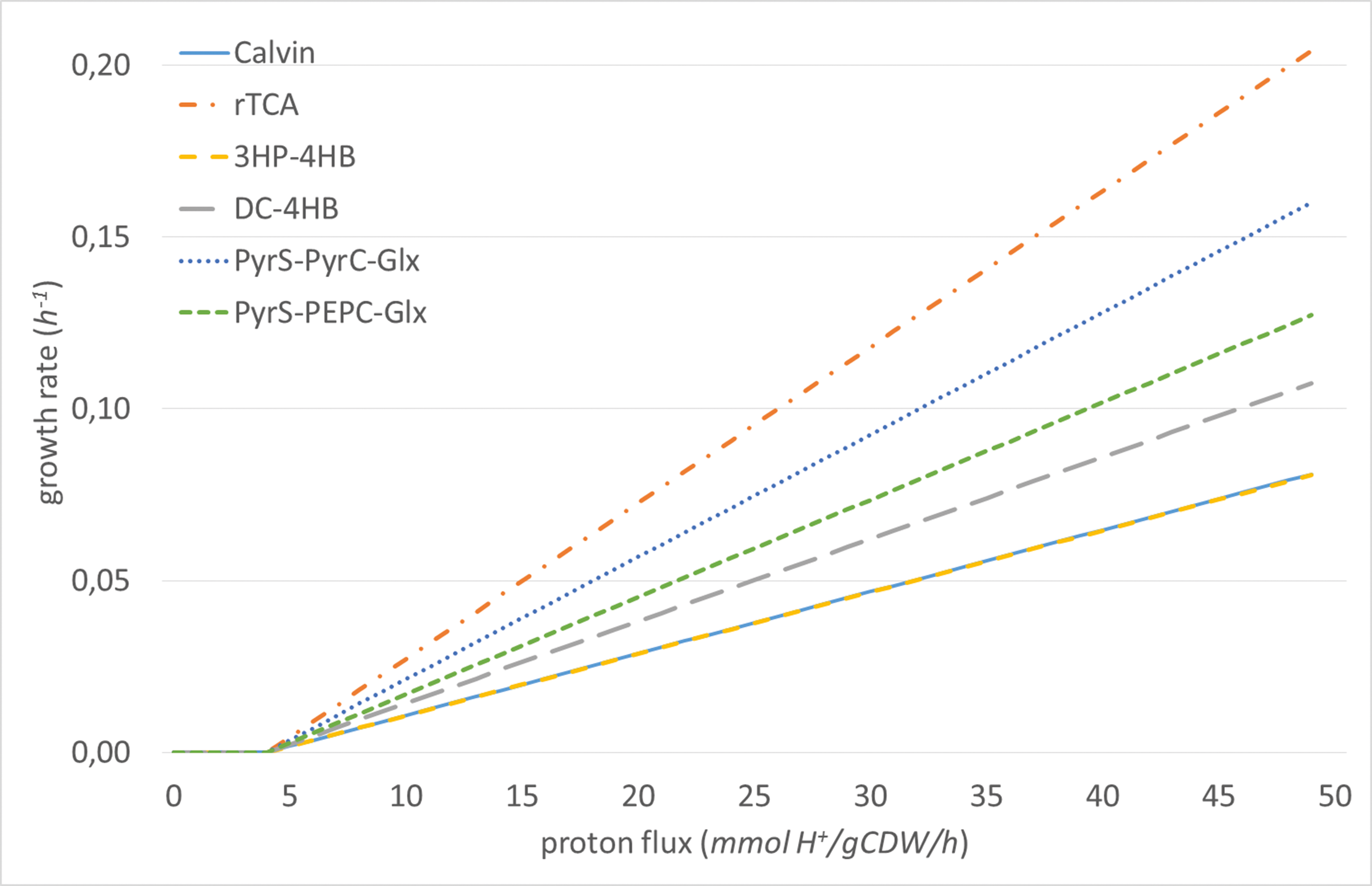

Supplement: S6 Fig — Predicted by FBA for different carbon fixation pathways with a NGAM of 1.00 mmol ATP/gCDW/h. PPR proton flux was varied between 0 and 50 mmol H+/gCDW/h. (TIF) [file pone.0157851.s006.tif]

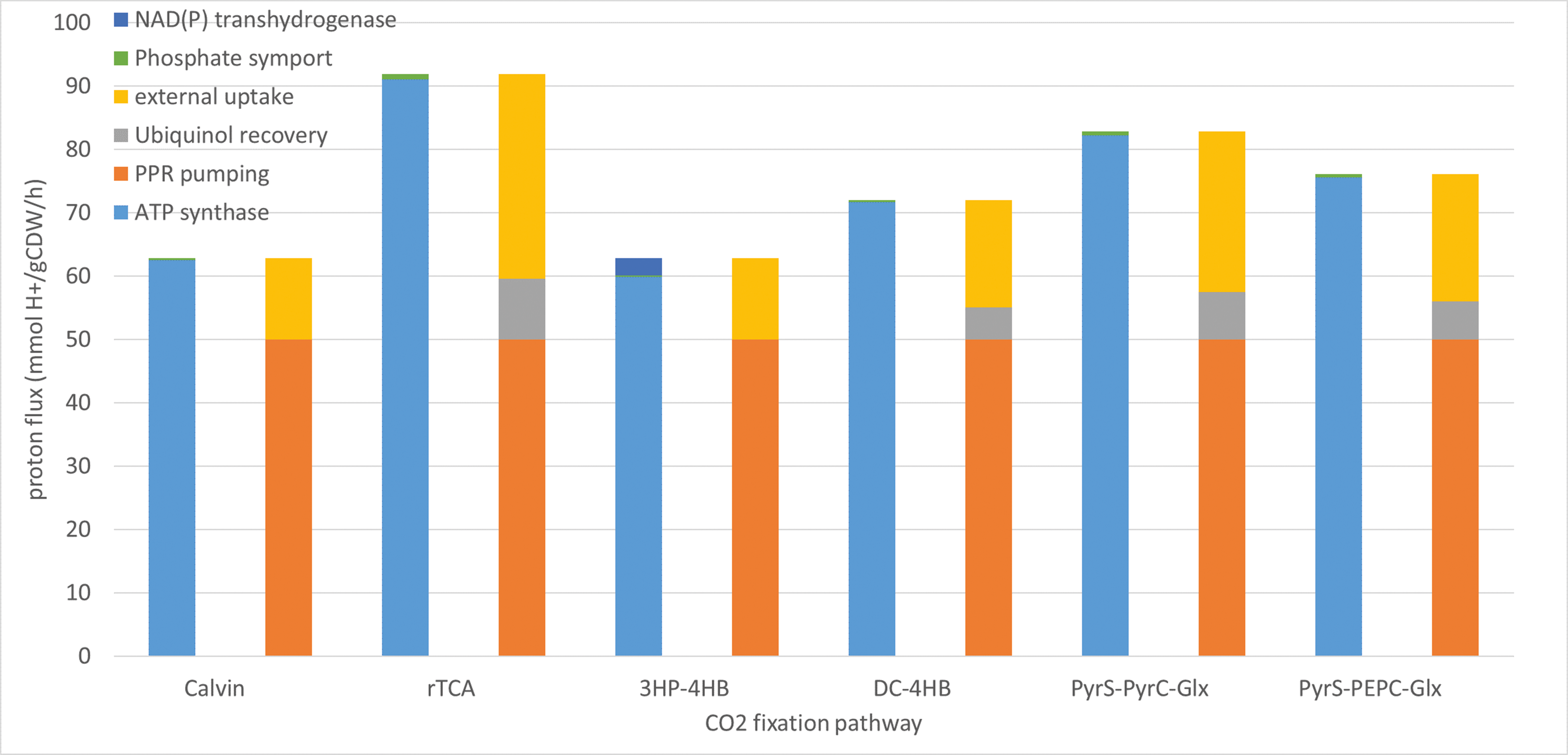

Supplement: S7 Fig — Major proton fluxes in and out of the cell during at a proton-pumping flux of 50 mmol H+/gCDW/h and a NGAM of 1.00 mmol ATP/gCDW/h (TIF) [file pone.0157851.s007.tif]

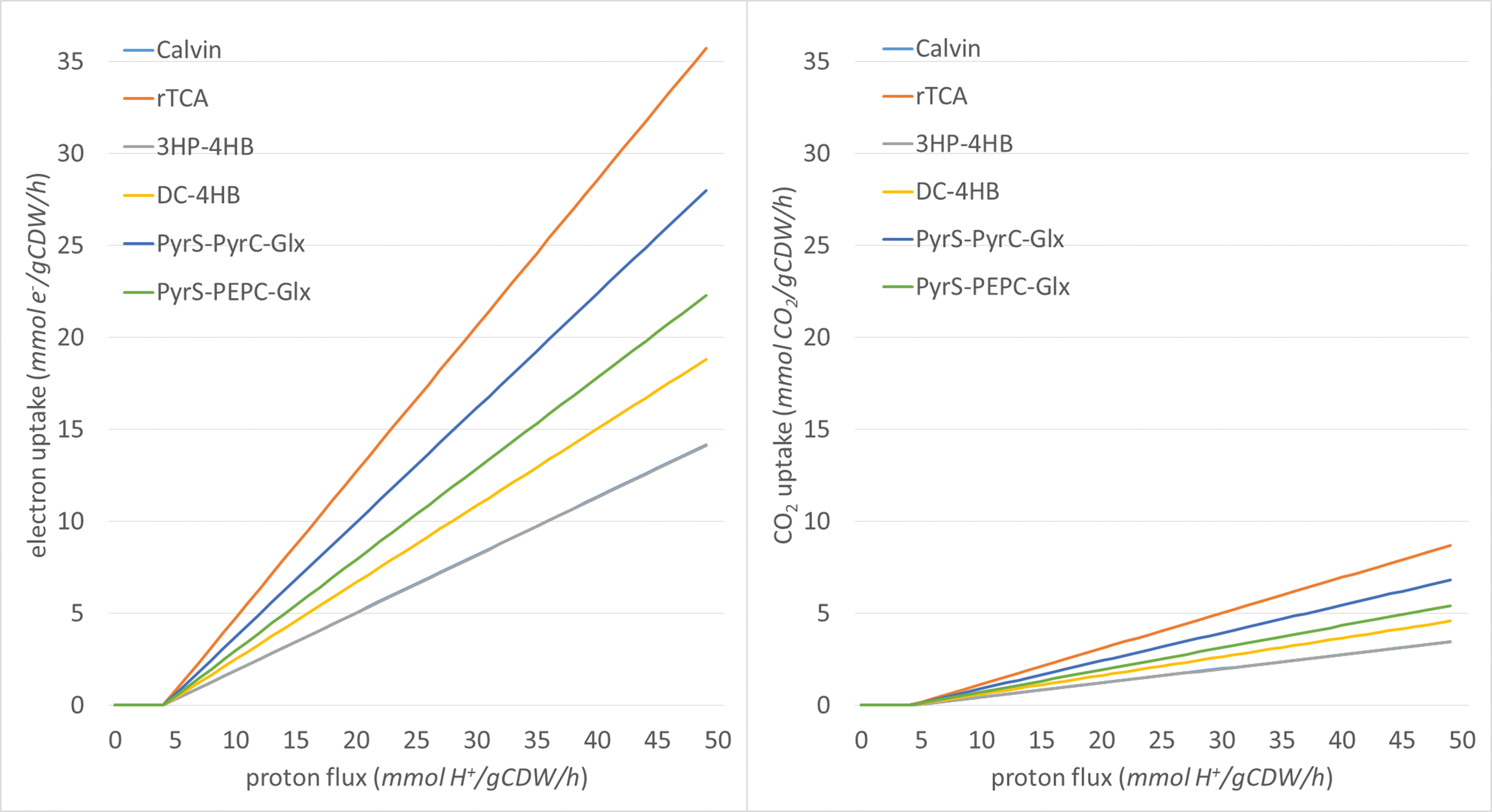

Supplement: S8 Fig — Proton pumping was varied between 0 and 50 mmol H+/gCDW/h. (TIF) [file pone.0157851.s008.tif]

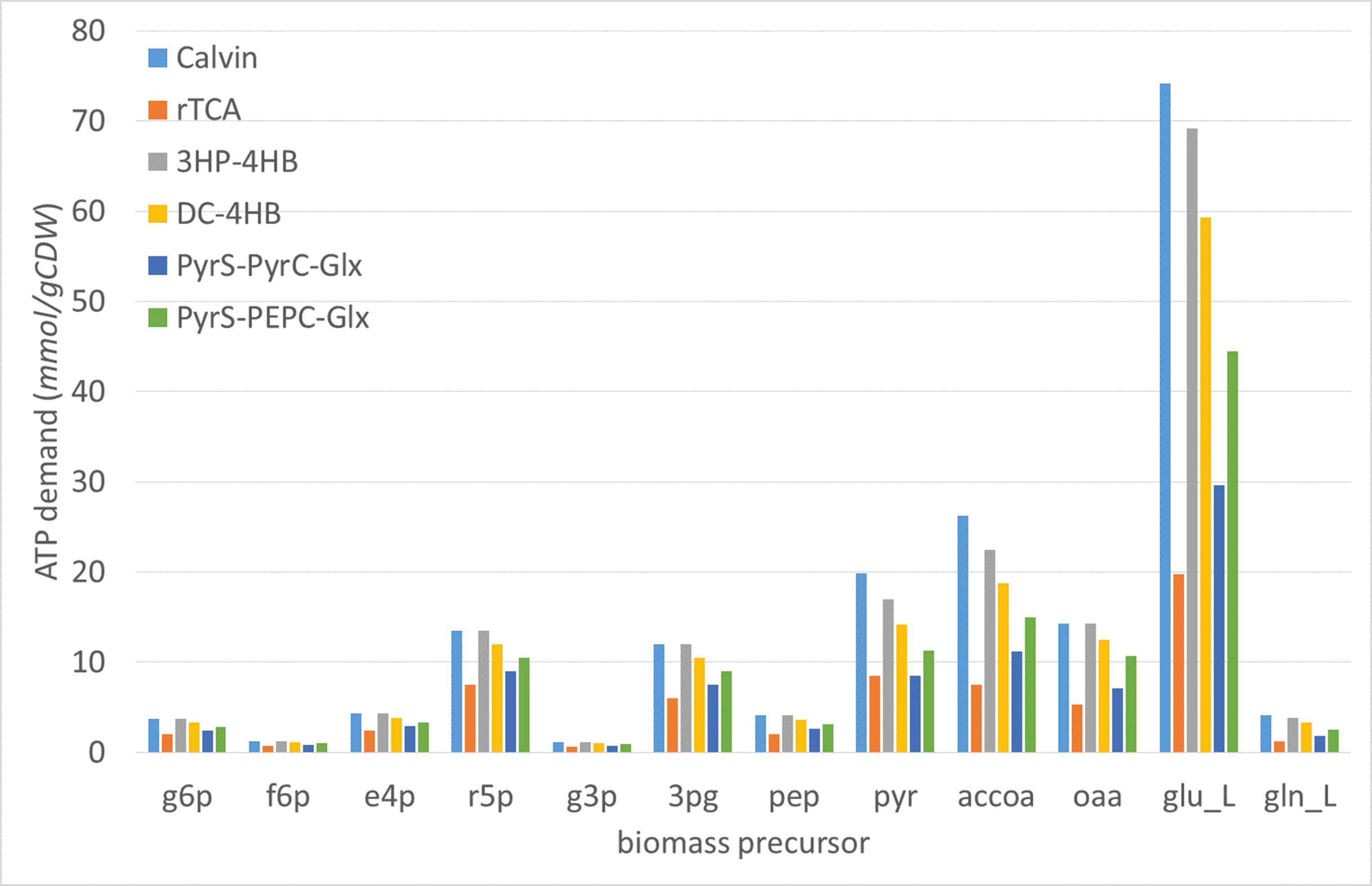

Supplement: S9 Fig — Biomass precursors are: D-glucose-6-phosphate (g6p), D-fructose-6-phosphate (f6p), D-erythrose-4-phosphate (e4p), α-D-ribose-5-phosphate (r5p), glyceraldehyde-3-phosphate (g3p), 3-phospho-D-glycerate (3pg), phosphoenolpyruvate (pep), pyruvate (pyr), acetyl-CoA (accoa), oxaloacetate (oaa), L-glutamate (glu_L) and L-glutamine (gln_L). (TIF) [file pone.0157851.s009.tif]

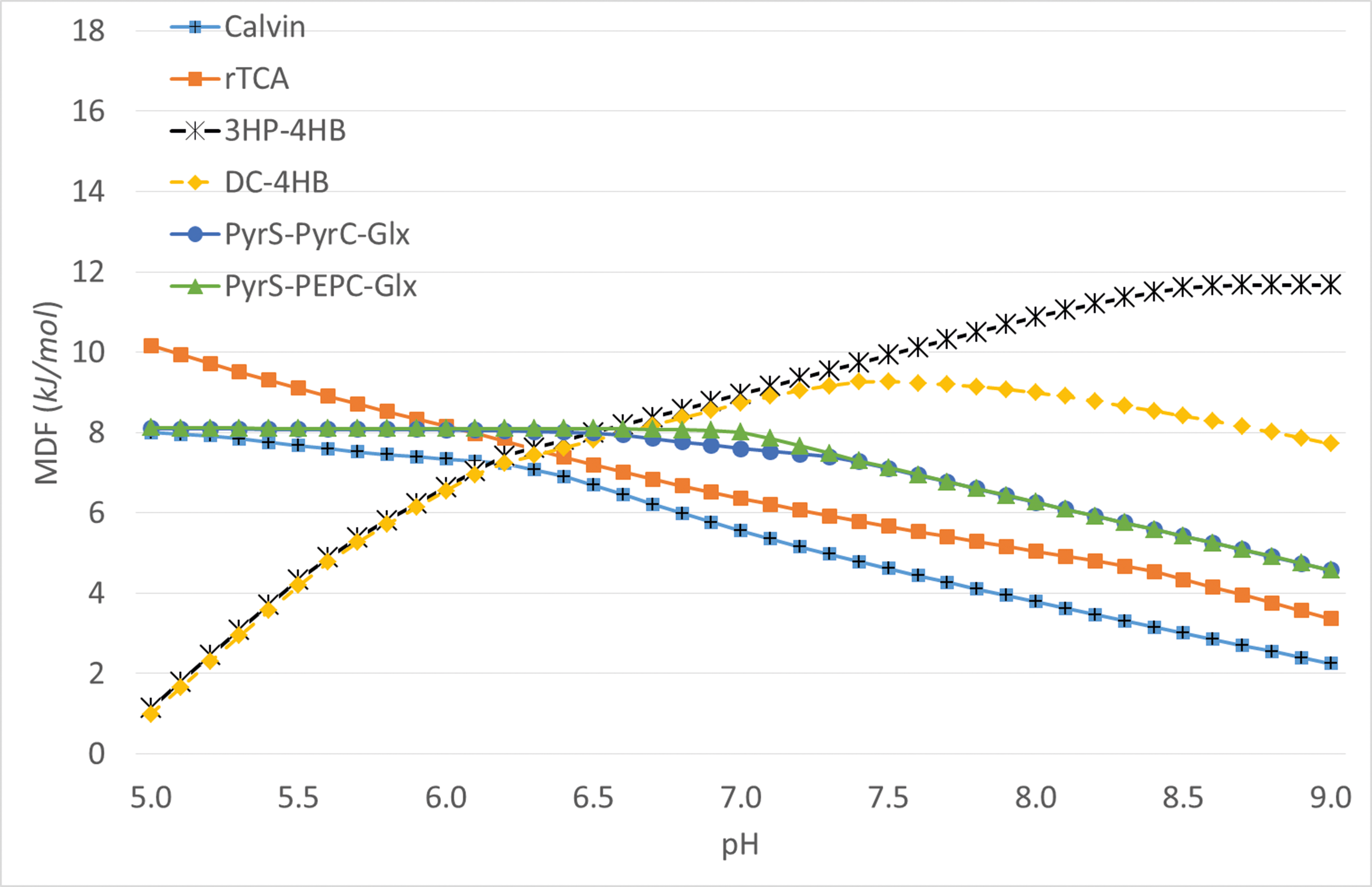

Supplement: S10 Fig — (TIF) [file pone.0157851.s010.tif]

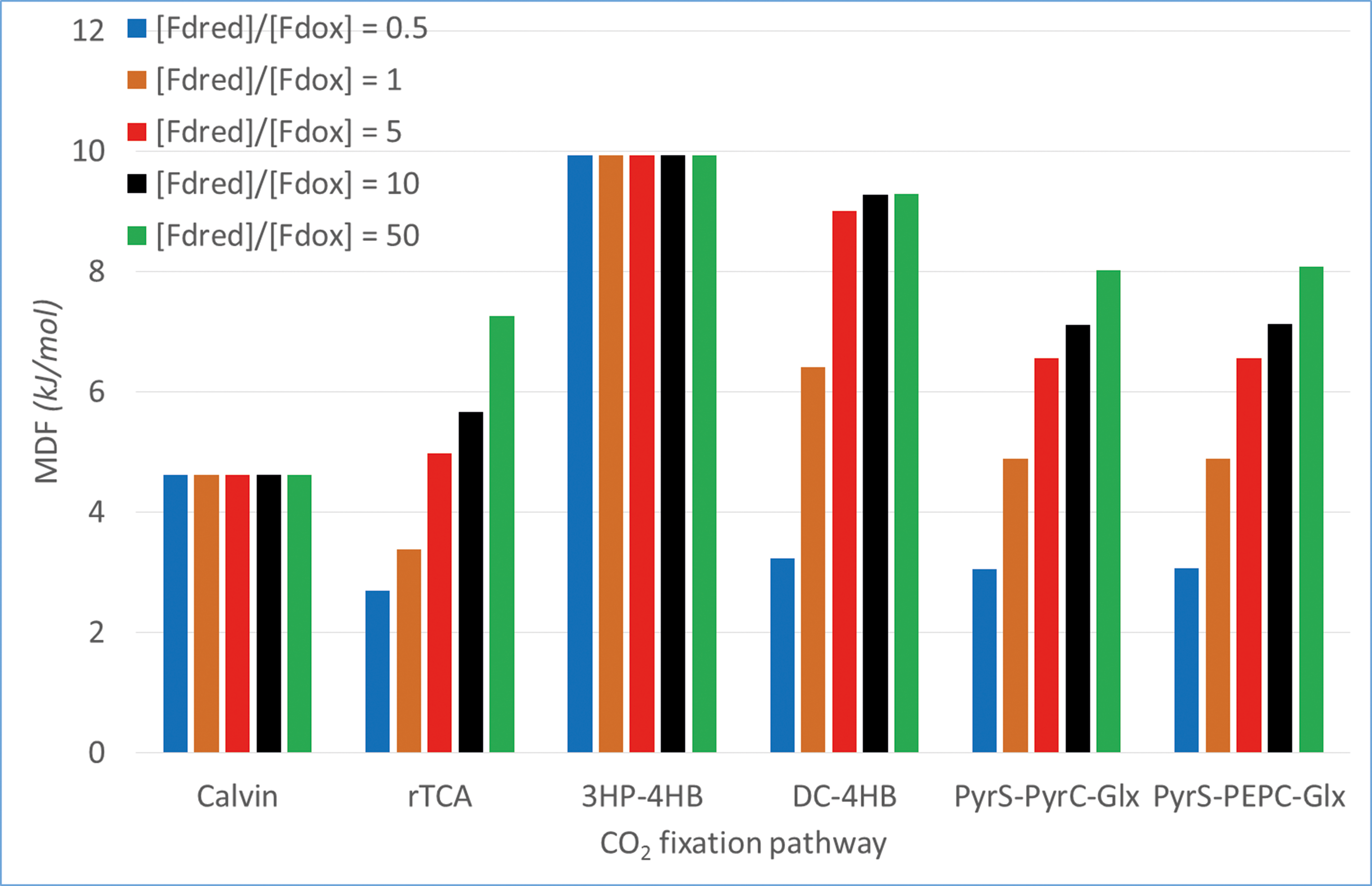

Supplement: S11 Fig — Bar labels indicate which reaction results in this MDF values, so which reaction forms the thermodynamic bottleneck. Bottleneck reactions are: glyceraldehyde-3-phosphate dehydrogenase (GAPD), isocitrate dehydrogenase (ICDHyr), 2-oxoglutarate:ferredoxin-oxidoreductase synthase (OGOR), succinyl-CoA synthetase (SUCOAS), pyruvate synthase (PYRS), (S)-malyl-CoA lyase (MCL), 3-hydroxyacyl-CoA dehydrogenase (HACD1), 3-hydroxyacyl-CoA dehydratase (ECOAH1). (TIF) [file pone.0157851.s011.tif]

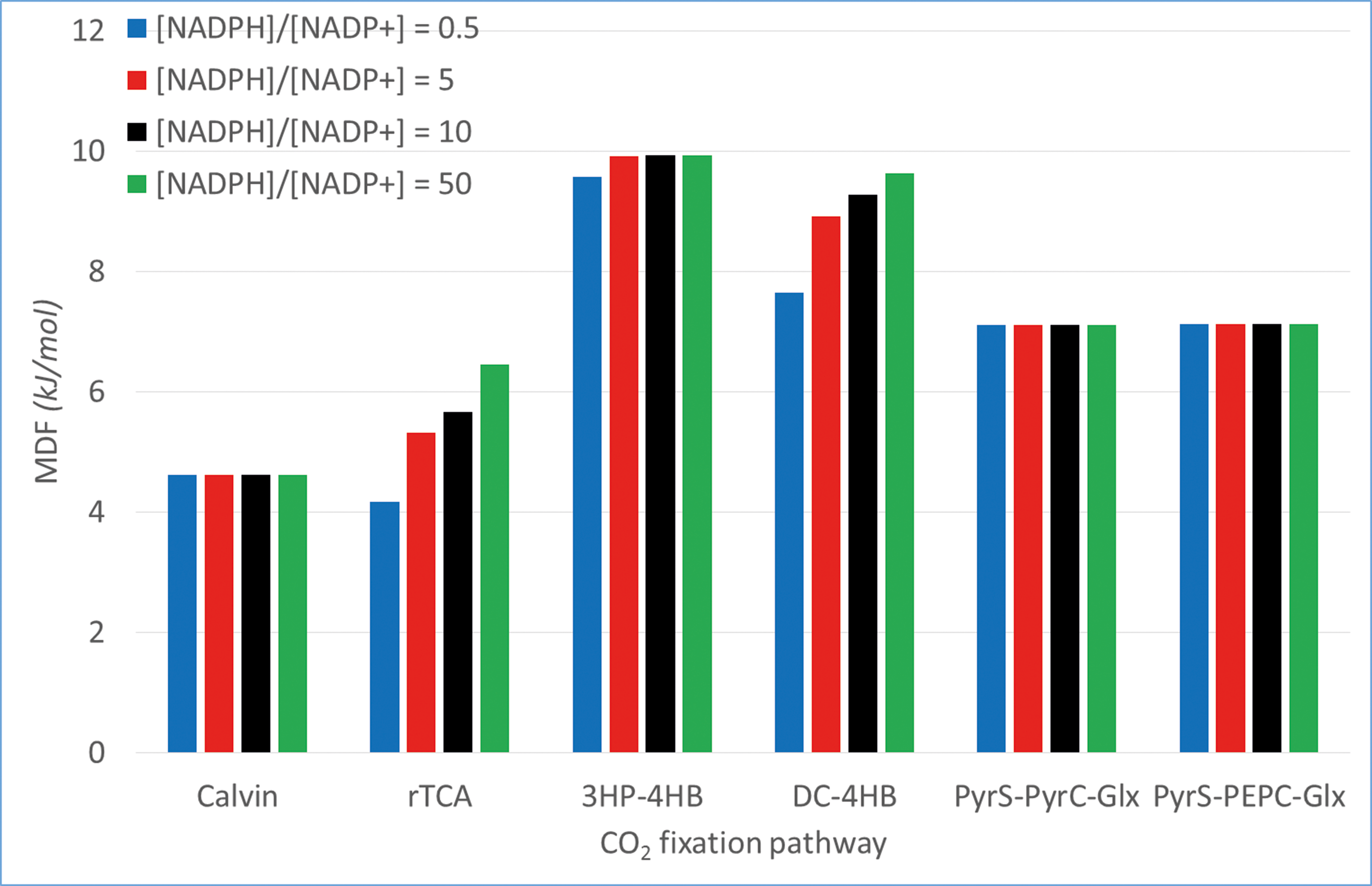

Supplement: S12 Fig — Bar labels indicate which reaction results in this MDF values, so which reaction forms the thermodynamic bottleneck. Bottleneck reactions are: glyceraldehyde-3-phosphate dehydrogenase (GAPD), isocitrate dehydrogenase (ICDHyr), succinyl-CoA synthetase (SUCOAS), pyruvate synthase (PYRS), 3-hydroxyacyl-CoA dehydrogenase (HACD1), acetyl-CoA C-acetyltransferase (ACACT1r). (TIF) [file pone.0157851.s012.tif]

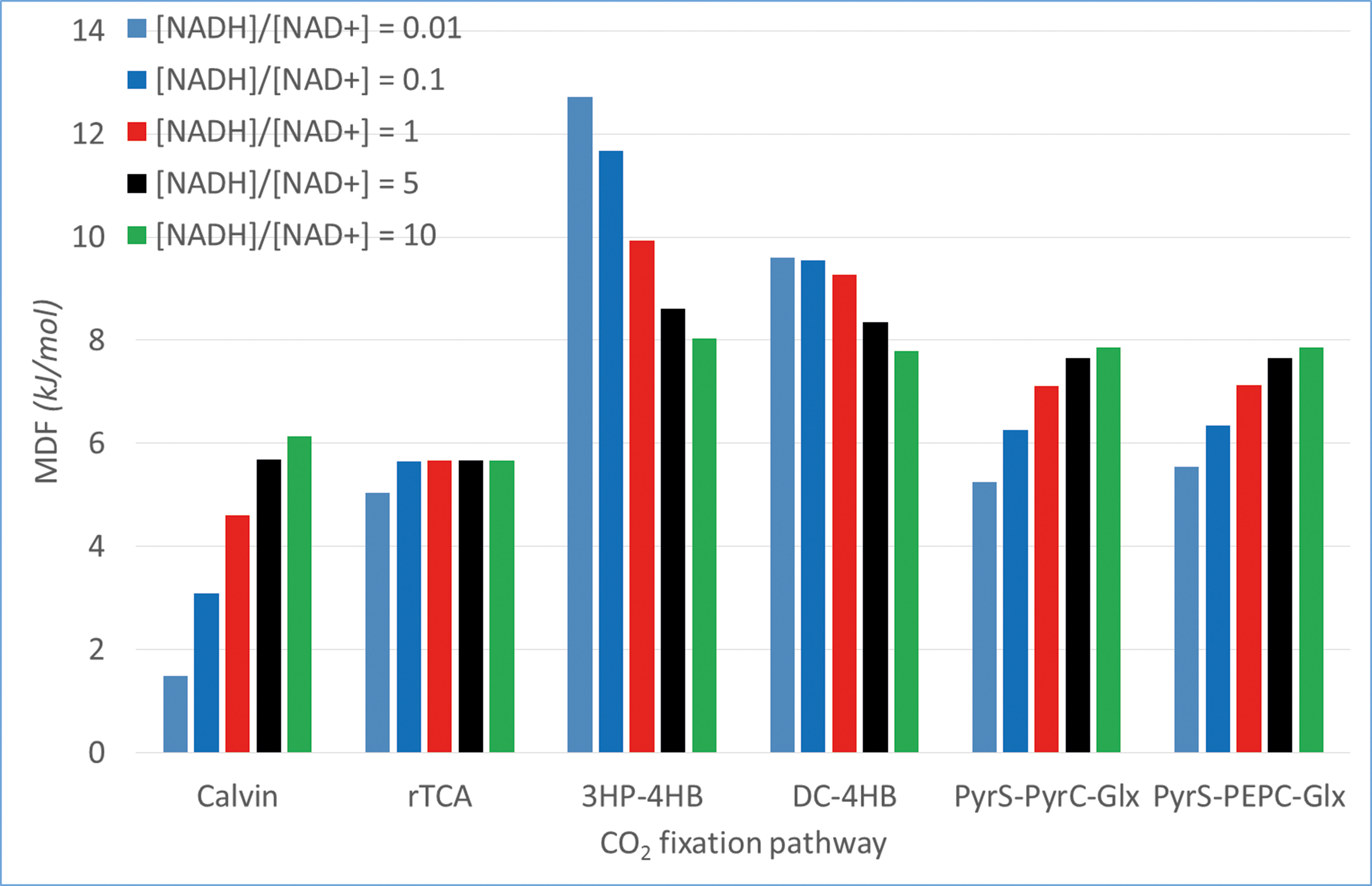

Supplement: S13 Fig — Bar labels indicate which reaction results in this MDF values, so which reaction forms the thermodynamic bottleneck. Bottleneck reactions are: glyceraldehyde-3-phosphate dehydrogenase (GAPD), ribose-5-phosphate isomerase (RPI), transketolase (TKT2), isocitrate dehydrogenase (ICDHyr), pyruvate synthase (PYRS), pyruvate carboxylase (PYRC), ATP citrate lyase (ACL), 3-hydroxyacyl-CoA dehydrogenase (HACD1), 3-hydroxyacyl-CoA dehydratase (ECOAH1). (TIF) [file pone.0157851.s013.tif]

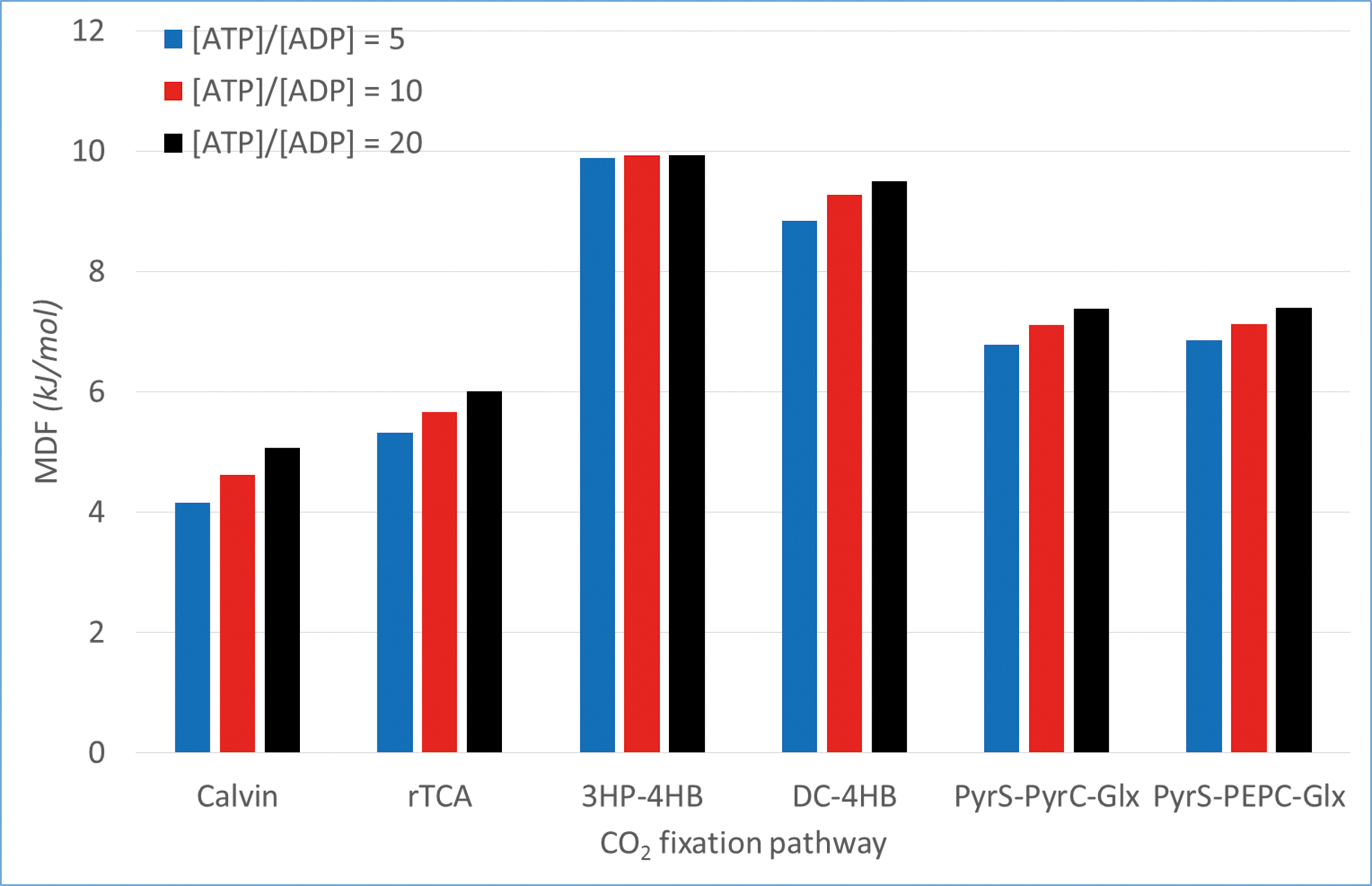

Supplement: S14 Fig — Bar labels indicate which reaction results in this MDF values, so which reaction forms the thermodynamic bottleneck. Bottleneck reactions are: ribose-5-phosphate isomerase (RPI), glyceraldehyde-3-phosphate dehydrogenase (GAPD), succinyl-CoA synthetase (SUCOAS), isocitrate dehydrogenase (ICDHyr), malate dehydrogenase (MDH), pyruvate synthase (PYRS), malyl-CoA synthetase (MTK), 3-hydroxyacyl-CoA dehydrogenase (HACD1), 4-hydroxybutyryl-CoA dehydratase (HB4COADH). (TIF) [file pone.0157851.s014.tif]

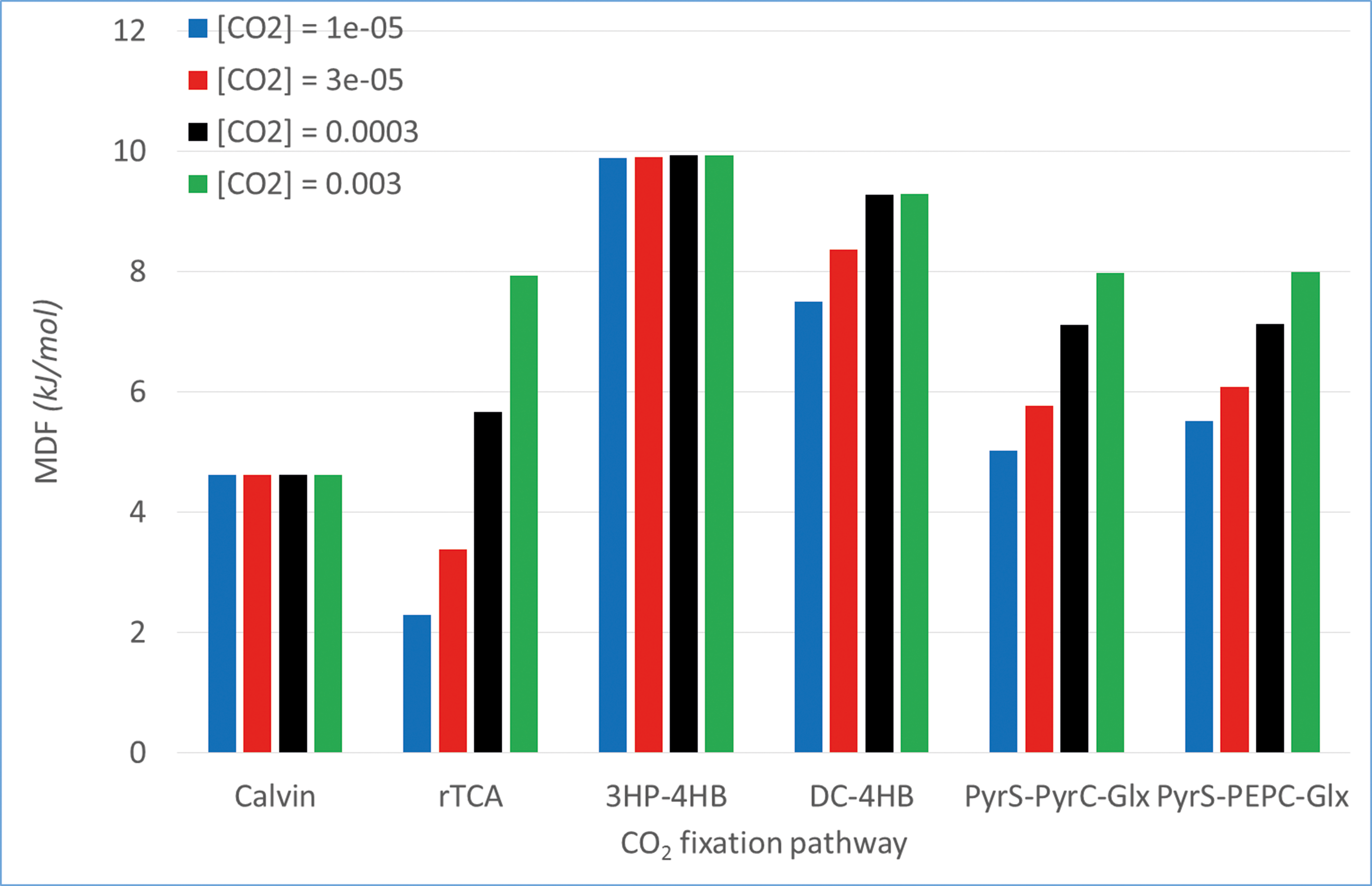

Supplement: S15 Fig — Bar labels indicate which reaction results in this MDF values, so which reaction forms the thermodynamic bottleneck. Bottleneck reactions are: glyceraldehyde-3-phosphate dehydrogenase (GAPD), ribulose-5-phosphate 3-epimerase (RPE), isocitrate dehydrogenase (ICDHyr), pyruvate synthase (PYRS), malate dehydrogenase (MDH), citrate hydrolyase (CITHL), 3-hydroxyacyl-CoA dehydrogenase (HACD1), 3-hydroxyacyl-CoA dehydratase (ECOAH1). (TIF) [file pone.0157851.s015.tif]

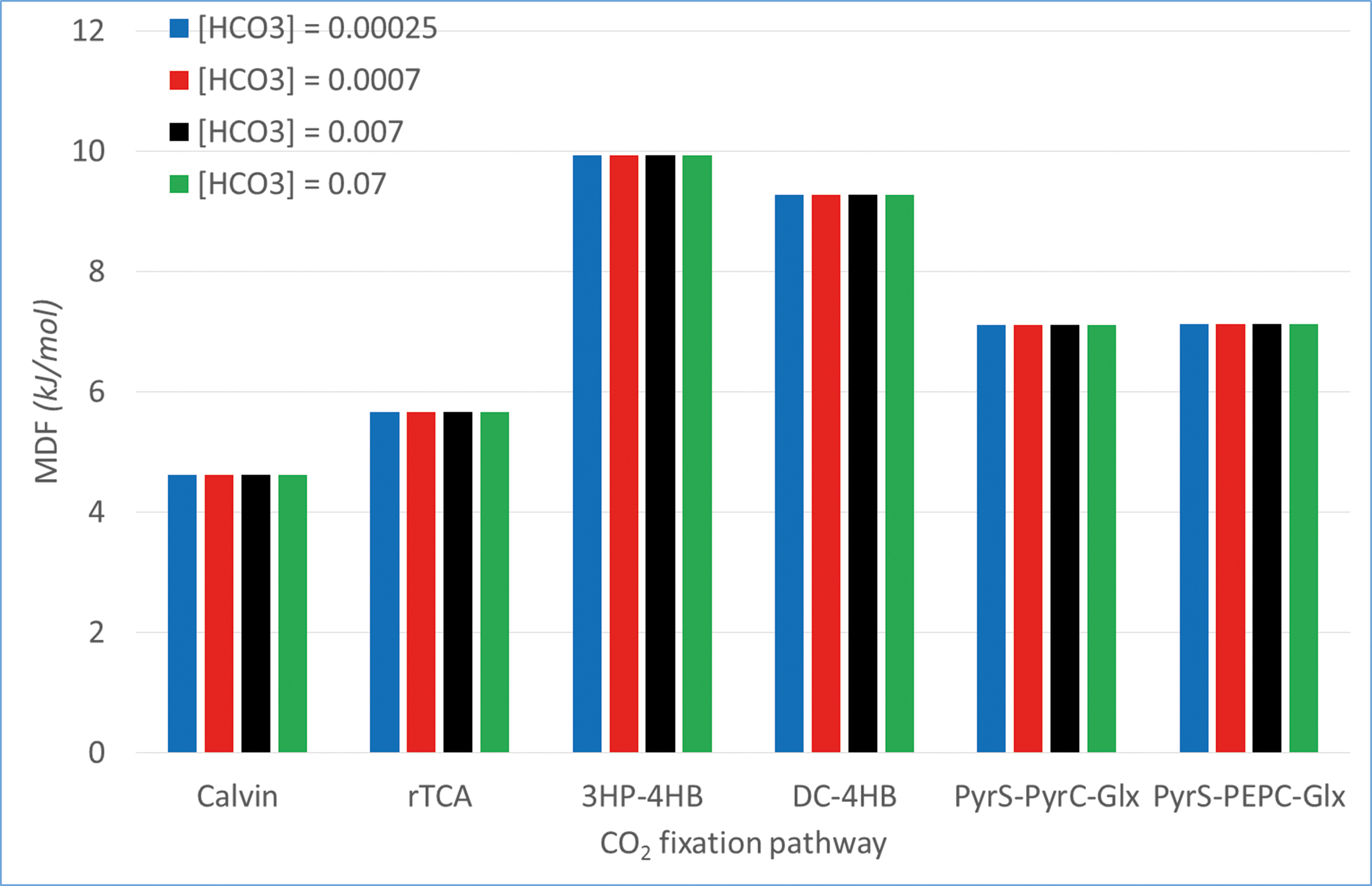

Supplement: S16 Fig — Bar labels indicate which reaction results in this MDF values, so which reaction forms the thermodynamic bottleneck. Bottleneck reactions are: glyceraldehyde-3-phosphate dehydrogenase (GAPD), isocitrate dehydrogenase (ICDHyr), malate dehydrogenase (MDH), (S)-malyl-CoA lyase (MCL), pyruvate synthase (PYRS), 3-hydroxyacyl-CoA dehydratase (ECOAH1), 3-hydroxyacyl-CoA dehydrogenase (HACD1). (TIF) [file pone.0157851.s016.tif]
