# Supplementary material for: Integrated In Silico Analysis of Pathway Designs for Synthetic Photo-Electro-Autotrophy
Source: PLoS One. 2016 Jun 23;11(6):e0157851. doi: 10.1371/journal.pone.0157851 (PMC4919048; doi:10.1371/journal.pone.0157851)
Supplement: S1 Text — (PDF) [file pone.0157851.s019.pdf]

S1 Text. Comparison of FBA results of core model versus genome-scale model

Here we compare the FBA results of the core model versus the full model. The *E. coli* core model is based on the *E. coli* genome-scale metabolic model AF1260 and consists only of the core metabolism of *E. coli* [31] (including glycolysis, pentose phosphate cycle, TCA cycle, glyoxylate shunt, respiration, etc.). To rule out a bias due to the use of the core model in our analysis, we included the reactions for the PPRs, the electron donor regeneration and the carbon fixation cycles in the genome-scale AF1260 model [33] and performed the FBA for the same constraints and modifications discussed in section 2.1-2.4 and 3.1. For the Calvin cycle and the 3HP-4HB cycle, it was necessary to additionally allow for succinate transport out of the cell. This byproduct formation is needed to have feasible growth conditions in these two scenarios for the genome-scale model, and not for the core model. Even though the resulting growth rates differ by up to 15%, the ranking of the different carbon fixation cycles does not change. Remarkably, the three better performing pathways, that is the rTCA cycle, the PyrS-PyrC-GLX and the PyrS-PEPC-GLX bicycle, show a lower growth rate in the genome-scale model compared to the core model, whereas the growth rates of the other three pathways increase when using the genome-scale model. This is due to the different composition of the biomass objective function. However, the rTCA cycle and the two synthetic bicycles still show a higher growth rate and combined with the results from the thermodynamic and pathway kinetics analysis, which do not depend on the choice of the model, the conclusions based on the core model are still valid. We believe, that the advantages of the core model, that is the better accessibility and the more general conclusions, overpower the more realistic but very specific representation when using the genome-scale metabolic model.

Carbon fixation pathway	Growth rate (hr^{-1})			
	low maintenance (1.00 mmol ATP/gCDW/h)		high maintenance (8.39 mmol ATP/gCDW/h)	
	Core model	Genome-scale model	Core model	Genome-scale model
Calvin cycle	0.083	0.090	0.030	0.032
rTCA cycle	0.209	0.173	0.071	0.062
PyrS-PyrC-Glx bicycle	0.164	0.149	0.058	0.053
PyrS-PEPC-Glx bicycle	0.130	0.115	0.047	0.041
3HP-4HB cycle	0.083	0.095	0.030	0.034
PyrS-PEPC-4HB cycle	0.110	0.113	0.039	0.041

Table A: Growth rates resulting from the FBA for the *E. coli* core model and the genome-scale model of *E. coli* for low and high non-growth associated maintenance.
