# Supplementary material for: Integrated In Silico Analysis of Pathway Designs for Synthetic Photo-Electro-Autotrophy
Source: PLoS One. 2016 Jun 23;11(6):e0157851. doi: 10.1371/journal.pone.0157851 (PMC4919048; doi:10.1371/journal.pone.0157851)
Supplement: S2 Text — (PDF) [file pone.0157851.s020.pdf]

Max-min driving force (update)

Elad Noor

March 14, 2016

In [1], we presented a thermodynamic-based pathway analysis technique, denoted Max-min Driving Force (MDF), which identifies those reactions within a pathway whose rates are constrained by low thermodynamic driving force. One of the sources of data required for performing MDF analysis, is the standard Gibbs free energies of each one of the reactions, in the specified pH and ionic strength levels ($\Delta_r G^\circ$). In [1] and in this work, we obtained the Gibbs energy data from the *Component Contribution* method [2], which estimates the values for the vast majority of enzyme-catalyzed reactions. This method is based on multivariate linear regression and provides both a mean and standard error of each estimated value. In the original implementation of the MDF algorithm [1], we ignored the standard errors. In this work and in [3], we improved the implementation by relaxing the thermodynamic constraints in the MDF linear program with uncertainty slack variables, which reflect the standard error of the *Component Contribution* estimates. This new implementation is now available freely on GitHub at https://github.com/eladnoor/component-contribution, under "scripts/max_min_driving_force.py".

1 Sampling from a Multivariate Gaussian

Consider a D-dimensional random variable with a Gaussian distribution of mean μ and covariance Σ.

If we have a 1-dimensional random Gaussian sampler, we can sample from the multivariate distribution by sampling D times and then stretching and rotating the vector according to Σ. Specifically, we define the square root of the covariance matrix as

$$\sqrt{\Sigma} = U \cdot \sqrt{S} \cdot U^\top \tag{1}$$

where where S is a diagonal real matrix and U is unitary which are given by the Singular Value Decomposition (SVD) of the covariance matrix, i.e. $\Sigma = U \cdot S \cdot U^\top$ (note that Σ is Hermitian and thus diagonalizable with real eigenvalues).

If $\forall i: \ y_i \sim \mathcal{N}(0, 1)$ and we define $z \equiv \mu + y \cdot \sqrt{\Sigma}$ then

$$z \sim \mathcal{N}(\mu, \Sigma) \tag{2}$$

2 Linear Programming with Multivariate Gaussian

The same approach can be applied for setting hard linear constraints on a the original variable in the context of linear programming. We define the auxiliary variable $y \in [-1, 1]^D$ replace the random variable by the expression $\mu + K \cdot y \cdot \sqrt{\Sigma}$, where K is a parameter of how loose we want the constraints to be (typically, we use the value 1).

This approach is easily applied to linear problems that utilize thermodynamic constraints, and use the standard Gibbs energies provided by Component Contribution. The vector of $\Delta_r G^\circ$ for a given problem should be constrained to: $\Delta_r G^\circ = \Delta_r G^\circ_{cc,\bar{X}} + 1 \cdot y \cdot \sqrt{\Sigma}_{cc,\bar{X}}$

References

[1] Elad Noor, Arren Bar-Even, Avi Flamholz, Ed Reznik, Wolfram Liebermeister, and Ron Milo. Pathway thermodynamics highlights kinetic obstacles in central metabolism. *PLoS Comput. Biol.*, 10, 2014.

[2] Elad Noor, S. Hulda Haraldsdóttir, Ron Milo, and T. Ronan M. Fleming. Consistent estimation of gibbs energy using component contributions. *PLoS Comput. Biol.*, 9, Jan 2013.

[3] Luca Gerosa, B. Bart R. van Rijsewijk, Dimitris Christodoulou, Karl Kochanowski, B. Thomas S. Schmidt, Elad Noor, and Uwe Sauer. Pseudo-transition analysis identifies the key regulators of dynamic metabolic adaptations from steady-state data. *Cell Syst.*, 1, 2015.
