# Supplementary material for: Integrated In Silico Analysis of Pathway Designs for Synthetic Photo-Electro-Autotrophy
Source: PLoS One. 2016 Jun 23;11(6):e0157851. doi: 10.1371/journal.pone.0157851 (PMC4919048; doi:10.1371/journal.pone.0157851)
Supplement: S3 Text — (PDF) [file pone.0157851.s021.pdf]

S3 Text. Presence and heterologous expression of carbon fixation enzymes in *E. coli*

Here we review native presence and successful expression of all required enzymes for the carbon fixation pathways in *E. coli.*

For the Calvin cycle two required non-native enzymes have already been functionally expressed *in vivo* in *E. coli*. Several groups successfully expressed the enzymes ribulose-1,5-bisphosphate carboxylase/oxygenase (RuBisCO) and phosphoribulokinase (PrkA), mostly originating from *Synechococcus* species in *E. coli* [1–6]. Probably a third enzyme of the Calvin cycle is not present in *E. coli* natively, i.e. sedoheptulose-1,7-bisphosphate aldolase. However, the native *E. coli* fructose-1,6-bisphosphatase may also have activity as an sedoheptulose-1,7-bisphosphate aldolase. For the native *E. coli* fructose-1,6-bisphosphatase aldolase it was shown that it also has sedoheptulose-1,7-bisphosphate aldolase activity [7]. The enzyme with both fructose-1,6-bisphosphatate aldolase and sedoheptulose-1,7-bisphosphate aldolase activity from *Bacillus methanolicus* has been heterologously expressed in *E. coli* and activity was shown *in vitro*, but not yet *in vivo* [8].

The rTCA cycle is for a large part already present in *E. coli*, as it has the oxidative citric acid cycle natively, which is basically the reverse of the rTCA cycle. However, for two irreversible steps of the citric acid cycle, heterologously expressed enzymes are needed. These two enzymes are a 2-oxoglutarate:ferredoxin oxidoreductase synthase (OGOR) and ATP-citrate lyase (ACL). Recently, ACL was functionally expressed in *E. coli* by overexpressing AclBA from *Chlorobium tepidum* [9]. Two OGOR enzymes from the thermophilic bacterium *Hydrogenobacter thermophilus* were expressed in *E. coli* and then purified and shown to be functional [10]. However, the activity was only demonstrated *in vitro* at 70 °C. So, *in vivo* functional expression of a mesophilic OGOR in *E. coli* has not been demonstrated so far. However, many mesophilic rTCA-harboring autotrophs exist, which can offer potential OGOR candidate genes for expression in *E. coli* [11].

Of the enzymes in the 3HP-4HB cycle, a large number is not present in *E. coli* natively. In the 3HP-4HB pathway malonyl-CoA is converted to R-methylmalonyl-CoA by four different enzymes. All these four enzymes have been functionally expressed in *E. coli* in an attempt to engineer the 3-hydroxypropionate bicycle, another natural carbon fixation pathway [12]. For the conversion of succinyl-CoA to 4-hydroxybutyrate another two enzymes need to be expressed heterologously. Both these enzymes were expressed successfully for 1,4-butanediol production in *E. coli* [13]. The next step in the pathway is the ATP-dependent conversion of 4-hydroxybutyrate to hydroxybutyryl-CoA; the enzyme catalyzing this conversion, 4-hydroxybutyryl-CoA synthetase, has not been expressed in *E. coli* so far. However, an alternative that has been used successfully in *E. coli* is 4-hydroxybutyryl-CoA transferase [13]. This reaction, however, is coupled to the conversion of acetyl-CoA to acetate instead of the phosphorylation by ATP. The generated acetate from the transferase could be recycled again to acetyl-CoA by native *E. coli* acetyl-CoA synthetase, which results in the same net ATP costs. Conversion of 4-hydroxobutyryl-CoA to crotonyl-CoA by 4-hydroxybutyryl-CoA dehydratase has, to our knowledge, not been achieved yet *in vivo* in *E. coli*, but the functional mesophilic enzyme from *Clostridium aminobutyricum* was purified after expression in *E. coli* [14]. The last three enzymes required for the conversion of crotonyl-CoA into acetyl-CoA are all natively present in *E. coli*, but also have been heterologously expressed from *Clostridium acetobutylicum* for butanol production [15]. In total, engineering the 3HP-4HB cycle would involve heterologous expression of eight enzymes in *E. coli*, the highest number for all analyzed pathways. Engineering of the complete 3HP-4HB cycle has to our knowledge not been tried yet in *E. coli*, however functional engineering of a small part of this cycle was already demonstrated for the thermophilic heterotroph *P. furiosus* [16]. For the synthetic 3HP-4HB variant, the PyrS-PEPC-4HB cycle, only 4 enzymes need to be introduced into *E. coli* for the conversion of succinyl-CoA to crotonyl-CoA.

For the synthetic PyrS-PEPC-Glx bicycle eleven enzymes are needed, of which three are non-native in *E. coli*. These three enzymes are all part of the so-called reverse glyoxylate shunt: malatethiokinase, malyl-CoA lyase and ACL, the latter is also part of the rTCA cycle. These three enzymes have been heterologously expressed together to form a functional reverse glyoxylate shunt in *E. coli* [9]. For another enzyme in the PyrS-PEPC-Glx bicycle: pyruvate synthase (PyrS), it was for a long time not clear if it is natively present in *E. coli* and what gene codes for this enzyme. However, PyrS activity has been detected already some time ago in soluble extracts of *E. coli* cultured under aerobic and anaerobic conditions [17] and later *ydbK* was identified as a hypothetical gene coding for PyrS [18]. This enzyme was identified as a pyruvate:flavodoxin oxidoreductase [19]. Overexpression of this gene in *E. coli*, gave indirect proof that ydbK may also use ferredoxin instead of flavodoxin for the reverse PyrS reaction [20,21]. So probably this *ydbK* gene can be overexpressed for a functional PyrS-PEPC-Glx

bicycle in *E. coli*. For the PyrS-PyrC-Glx bicycle the same heterologous enzymes as for the PyrS-PEPC-Glx variant are required. In addition, this pathway requires heterologous expression of a PyrC enzyme in *E. coli*. Heterologous expression of PyrC for succinate production in *E. coli* has been demonstrated [22,23]. So, for both synthetic pathways all required enzymes have already been expressed in *E. coli* before.

1. Parikh MR, Greene DN, Woods KK, Matsumura I. Directed evolution of RuBisCO hypermorphs through genetic selection in engineered *E.coli*. Protein Eng Des Sel. 2006;19: 113–119. doi:10.1093/protein/gzj010
2. Greene DN, Whitney SM, Matsumura I. Artificially evolved Synechococcus PCC6301 Rubisco variants exhibit improvements in folding and catalytic efficiency. Biochem J. 2007;404: 517–524. doi:10.1042/BJ20070071
3. Mueller-Cajar O, Morell M, Whitney SM. Directed evolution of Rubisco in Escherichia coli reveals a specificity-determining hydrogen bond in the form II enzyme. Biochemistry. 2007;46: 14067–14074. doi:10.1021/bi700820a
4. Mueller-Cajar O, Whitney SM. Evolving improved *Synechococcus* Rubisco functional expression in *Escherichia coli*. Biochem J. 2008;414: 205–214. doi:10.1042/BJ20080668
5. Zhuang Z-Y, Li S-Y. Rubisco-based engineered *Escherichia coli* for in situ carbon dioxide recycling. Bioresour Technol. 2013;150: 79–88. doi:10.1016/j.biortech.2013.09.116
6. Gong F, Liu G, Zhai X, Zhou J, Cai Z, Li Y. Quantitative analysis of an engineered CO_2-fixing *Escherichia coli* reveals great potential of heterotrophic CO_2 fixation. Biotechnol Biofuels. 2015;8. doi:10.1186/s13068-015-0268-1
7. Nakahigashi K, Toya Y, Ishii N, Soga T, Hasegawa M, Watanabe H, et al. Systematic phenome analysis of Escherichia coli multiple-knockout mutants reveals hidden reactions in central carbon metabolism. Mol Syst Biol. 2009;5. doi:10.1038/msb.2009.65
8. Stolzenberger J, Lindner SN, Persicke M, Brautaset T, Wendisch VF. Characterization of fructose 1,6-bisphosphatase and sedoheptulose 1,7-bisphosphatase from the facultative ribulose monophosphate cycle methylotroph Bacillus methanolicus. J Bacteriol. 2013;195: 5112–5122. doi:10.1128/JB.00672-13
9. Mainguet SE, Gronenberg LS, Wong SS, Liao JC. A reverse glyoxylate shunt to build a non-native route from C4 to C2 in *Escherichia coli*. Metab Eng. Elsevier; 2013;16: 116–127. doi:10.1016/j.ymben.2013.06.004
10. Yamamoto M, Arai H, Ishii M, Igarashi Y. Characterization of two different 2-oxoglutarate:ferredoxin oxidoreductases from *Hydrogenobacter thermophilus* TK-6. Biochem Biophys Res Commun. 2003;312: 1297–1302. doi:10.1016/j.bbrc.2003.11.078
11. Berg IA. Ecological aspects of the distribution of different autotrophic CO_2 fixation pathways. Appl Environ Microbiol. 2011;77: 1925–1936. doi:10.1128/AEM.02473-10
12. Mattozzi MD, Ziesack M, Voges MJ, Silver PA, Way JC. Expression of the sub-pathways of the *Chloroflexus aurantiacus* 3-hydroxypropionate carbon fixation bicycle in *E. coli*: Toward horizontal transfer of autotrophic growth. Metab Eng. Elsevier; 2013;16: 130–139. doi:10.1016/j.ymben.2013.01.005

13. Yim H, Haselbeck R, Niu W, Pujol-Baxley C, Burgard A, Boldt J, et al. Metabolic engineering of *Escherichia coli* for direct production of 1, 4-butanediol. Nat Chem Biol. Nature Publishing Group; 2011;7: 445–452. doi:10.1038/nchembio.580
14. Gerhardt A, Çinkaya I, Linder D, Huisman G, Buckel W. Fermentation of 4-aminobutyrate by Clostridium aminobutyricum: cloning of two genes involved in the formation and dehydration of 4-hydroxybutyryl-CoA. Arch Microbiol. 2000;174: 189–199. doi:10.1007/s002030000195
15. Inui M, Suda M, Kimura S, Yasuda K, Suzuki H, Toda H, et al. Expression of Clostridium acetobutylicum butanol synthetic genes in Escherichia coli. Appl Microbiol Biotechnol. 2008;77: 1305–1316. doi:10.1007/s00253-007-1257-5
16. Keller MW, Schut GJ, Lipscomb GL, Menon AL, Iwuchukwu IJ, Leuko TT, et al. Exploiting microbial hyperthermophilicity to produce an industrial chemical, using hydrogen and carbon dioxide. Proc Natl Acad Sci U S A. 2013;110: 5840–5845. doi:10.1073/pnas.1222607110
17. Blaschkowski HP, Knappe J, Ludwig-Festl M, Neuer G. Routes of flavodoxin and ferredoxin reduction in Escherichia coli. Eur J Biochem. 1982;123: 563–569. doi:10.1111/j.1432-1033.1982.tb06569.x
18. Reed JL, Vo TD, Schilling CH, Palsson BØ. An expanded genome-scale model of *Escherichia coli* K-12 (iJR904 GSM/GPR). Genome Biol. 2003;4. doi:10.1186/gb-2003-4-9-r54
19. Nakayama T, Yonekura S, Yonei S. *Escherichia coli* pyruvate : flavodoxin oxidoreductase , YdbK - regulation of expression and biological roles in protection against oxidative stress. Genes Genet Syst. 2013;88: 175–188.
20. Eremina NS, Yampolskaya TA, Altman IB, Mashko S V, Stoynova N V. Overexpression of ydbK-encoding putative pyruvate synthase improves L-valine production and aerobic growth on ethanol media by an *Escherichia coli* strain carrying an oxygen-resistant alcohol dehydrogenase. J Microb Biochem Technol. 2010;02: 77–83. doi:10.4172/1948-5948.1000028
21. Akhtar MK, Jones PR. Construction of a synthetic YdbK-dependent pyruvate:H2 pathway in *Escherichia coli* BL21(DE3). Metab Eng. 2009;11: 139–147. doi:10.1016/j.ymben.2009.01.002
22. Gokarn RR, Eiteman MA, Altman E. Expression of pyruvate carboxylase enhances succinate production in *Escherichia coli* without affecting glucose uptake. Biotechnol Lett. Springer; 1998;20: 795–798. doi:10.1023/B:BILE.0000015925.52287.1f
23. Wang Q, Wu C, Chen T, Chen X, Zhao X. Expression of galactose permease and pyruvate carboxylase in *Escherichia coli* ptsG mutant increases the growth rate and succinate yield under anaerobic conditions. Biotechnol Lett. 2006;28: 89–93. doi:10.1007/s10529-005-4952-2
